# Microplastics in the Soil at Sub‐Toxic Concentrations Cause Metabolic Changes Decreasing Fungal Pathogen Susceptibility in *Arabidopsis thaliana*

**DOI:** 10.1111/ppl.70312

**Published:** 2025-06-09

**Authors:** Marco Dainelli, Costanza Cicchi, Ivan Baccelli, Stéphanie Boutet, Ilaria Colzi, Andrea Coppi, Simone Luti, Sara Pignattelli, Susanna Pollastri, Francesco Loreto, Luigia Pazzagli, Massimiliano Corso, Cristina Gonnelli

**Affiliations:** ^1^ Department of Biology University of Florence Florence Italy; ^2^ Department of Biomedical Experimental and Clinical Sciences Mario Serio, University of Florence Florence Italy; ^3^ Institute for Sustainable Plant Protection (IPSP), National Research Council of Italy (CNR) Sesto Fiorentino Italy; ^4^ Université Paris‐Saclay, INRAE, AgroParisTech, Institut Jean‐Pierre Bourgin for Plant Sciences (IJPB) Versailles France; ^5^ Institute of Bioscience and Bioresources (IBBR), National Research Council (CNR) Sesto Fiorentino Italy; ^6^ Department of Biology University of Naples Federico II Naples Italy

**Keywords:** *Arabidopsis*, *Botrytis*, microplastics, PAMP, specialised metabolism

## Abstract

To unravel the complex interactions between microplastics (MPs), plants, and pathogens, *Arabidopsis thaliana* plants were grown for 3 weeks in soils containing polyethylene terephthalate (PET) or polyvinyl chloride (PVC) MPs (0.2% and 0.5% w/w), and leaves were then exposed to the PAMP (Pathogen‐Associated Molecular Pattern) protein cerato‐platanin (CP) or *Botrytis cinerea* conidia. PET caused a stimulation of stomatal conductance, and PVC decreased the aboveground biomass of *A. thaliana* plants. PVC (0.2%) triggered a primed state in *A. thaliana* , enhancing its response to *B. cinerea* infection and cerato‐platanin. This was demonstrated by decreased lesion size, enhanced ROS generation, and elevated camalexin synthesis following PAMP elicitation, and increased levels of defensive isothiocyanate and phenylpropanoid metabolites. Our results indicate that MPs also affect soil structure, ionome balance, and specialised metabolite accumulation. However, MPs did not provide an unambiguous response, underscoring challenges in formulating a model of plant response to MPs when exposed to pathogens.

## 1 Introduction

Since Thompson et al. (2004) coined the term “microplastics” in 2004 (MPs, size < 5 mm; a list of abbreviations used in this manuscript is provided in Table S1), the investigations on the effects of these tiny particles on the environment and organisms have proliferated (Roy et al. 2023). Attention was focused mainly on seas and oceans at first (Yang and Gao 2022), probably because of the hype caused by the Pacific Garbage Patch, an enormous and controversial “trash vortex”, mainly made by plastics, between Hawaii and California (Kaiser 2010). However, plastic pollution is not limited to marine systems, and no part of the biosphere can be considered plastic‐free. In the atmosphere, non‐visible particles are abundant and can have negative impacts on different living organisms (see, e.g., Enyoh et al. 2019; Falsini et al. 2022; Prata 2018). All MPs are of terrestrial origin, and the soil is the environmental compartment most polluted by these contaminants: MP concentrations can reach up to 7% of soil weight near industrial areas (de Souza Machado et al. 2019). The major sources of MPs in soils are wastes of plastic mulch films used in agriculture. Other remarkable inputs of MPs are organic fertilisers, irrigation waters, sewage sludge, biosolids, atmospheric deposition, and so on (Cusworth et al. 2024; Ng et al. 2018; Okeke et al. 2022; Uwamungu et al. 2022). MPs are hazardous to soil health, and thus to all soil‐living organisms, since they alter soil physical structure (e.g., porosity) and chemical and biological properties (Iqbal et al. 2023; Mondol et al. 2024; Zhang et al. 2022).

Plants are inevitably affected by this new class of pollutants since plants are sessile organisms and cannot escape all environmental challenges during their lifespan (Weng et al. 2021). A wide variety of negative effects caused by MPs on plants has already been reported and summarised in extensive reviews (e.g., Gan et al. 2023; Hu et al. 2024; Iqbal et al. 2023; Jia et al. 2023; Wang, Luo et al. 2023; Zantis et al. 2023). However, plant response to MPs is greatly variable, depending on multifaceted MP characteristics and specific plant capacity to interact with MPs (Karalija et al. 2022). Moreover, stressors seldom come alone in natural environments, and plants are always exposed to a combination of them (Xu et al. 2023; Zantis et al. 2023). Joint exposure of plants to MPs and other stressful conditions has started to be studied (Tourinho et al. 2023). This is essential for a correct understanding of MP hazardousness and/or toxicity, since, in general, combined stressors may have more severe effects on plants (Xu et al. 2023). For example, joint treatments with MPs and trace metals, especially cadmium and lead, may alter element bioavailability in environmental matrices (Abbasi et al. 2020; Huang et al. 2023; Wang et al. 2024) and aggravate their toxic effects on plants (Jia et al. 2022; Wang, Meng, et al. 2023). Often, MPs magnify the impacts of other stresses on plant organisms and communities, such as in the case of acid rain (Pignattelli et al. 2021), drought (Lozano and Rillig 2020) and heat (Maity et al. 2024).

Biotic stressors are a main cause of global agricultural yield losses (Khan et al. 2021), and plant communities are expected to be increasingly exposed to dangerous diseases in the current climate change scenario (Surówka et al. 2020). Foyer et al. (2016) demonstrated that various plant defence responses against abiotic stressors (e.g., reduced growth, redox regulation, production of specialised metabolites) could positively influence resistance to aphid infestation. Furthermore, the simultaneous presence of environmental contaminants, other than plastics, and biotic stress agents has been reported to possibly lead to cross‐tolerance (Poschenrieder et al. 2006). MPs are abundant in both natural environments and agroecosystems (Ng et al. 2018; Zhang et al. 2022); however, to the best of our knowledge, only two recent studies have investigated the combination of MPs with biotic stress, both suggesting higher plant susceptibility to the stressors (Cao et al. 2024; Bouaicha et al. 2024).

In order to shed light on the potential impact of MPs on plant‐pathogen interaction, the model plant *Arabidopsis thaliana* was grown in the presence of two widely diffused types of MPs, PET and PVC, at two environmentally relevant concentrations. We explored whether MPs: (i) affected plant resistance to *Botrytis cinerea*, a generalist fungal pathogen (Bi et al. 2023); (ii) influenced the production of antimicrobial compounds (i.e., phytoalexins) and reactive oxygen species (ROS) after exposure to cerato‐platanin (CP), a fungal protein with well‐known PAMP activity (Luti et al. 2016); (iii) altered plant specialised metabolism, thus contributing to explain possible interactions with biotic stress.

## 2 Materials and Methods

### 2.1 Plant Growth, Experimental Conditions and Biometric Traits

Microplastic soil pollution was simulated in the laboratory using previously standardised methodologies (Colzi et al. 2022; Dainelli et al. 2023) with slight modifications. PET and PVC MPs in powder form were purchased from Sigma‐Aldrich (Sigma‐Aldrich, quality level 100, semi‐crystalline, CAS‐number 25038‐59‐9 and 9002–86–2). The powders were further sieved through a 100 μm stainless steel mesh, obtaining particles with an average diameter of 40–50 μm. Polymer density was around 1.4 g cm^−3^ for both PET and PVC. These types of plastic were chosen because of their large presence in natural and agricultural soils (Büks and Kaupenjohann 2020; Tian et al. 2022). They were directly added to the substrate without further treatment at two environmentally realistic concentrations that are commonly used for testing in controlled conditions (Chen, Li, et al. 2022; Chen, Feng, et al. 2022; Wang, Luo, et al. 2023), that is, 0.2% and 0.5% MPs/dry soil (w/w), corresponding to 0.04 and 0.1 g of MPs per 20 g of dry soil, respectively.

**FIGURE 1:**
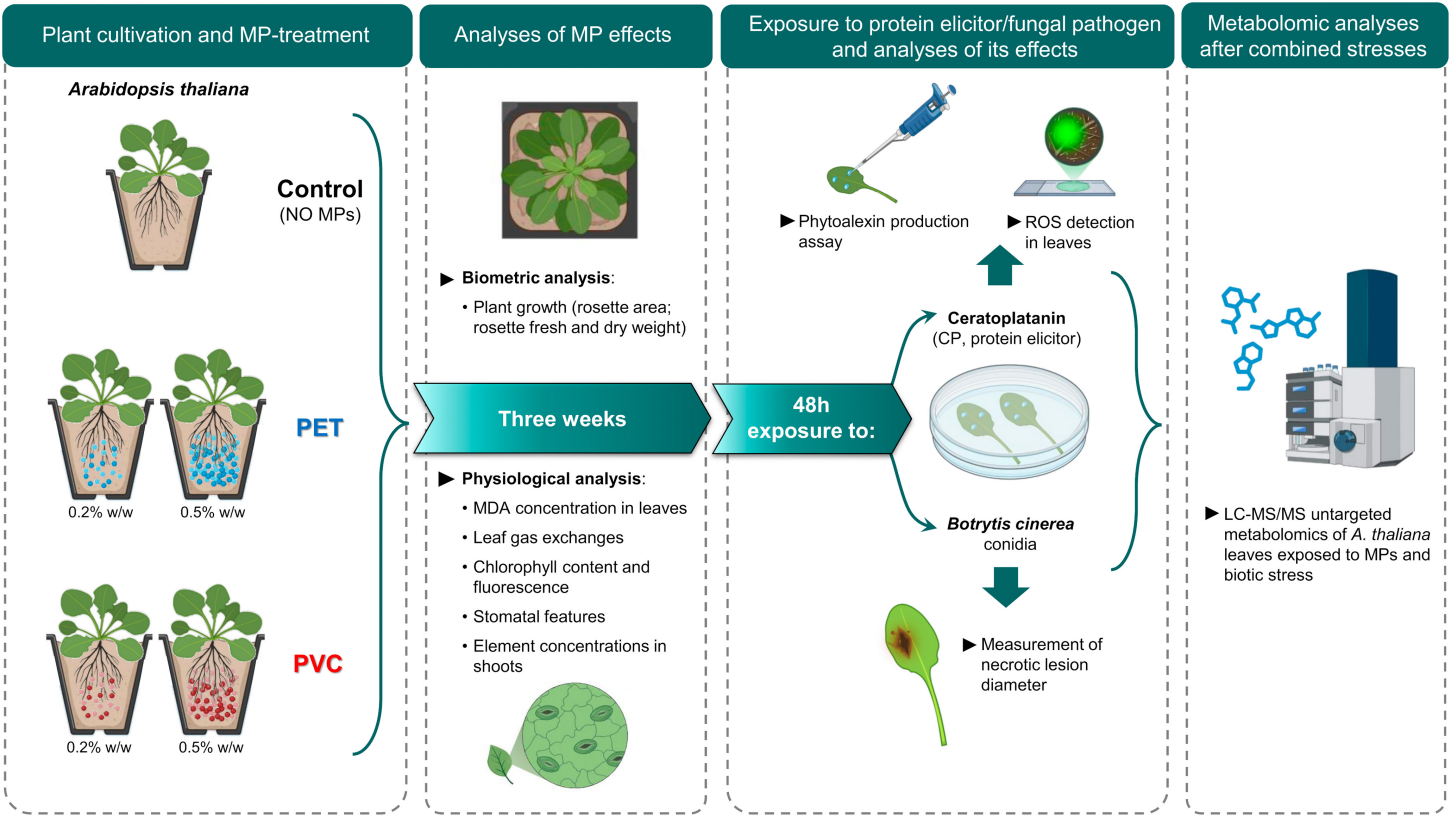
Graphical representation of the experimental setup and the subsequent analyses performed.

Five different treatment conditions were applied: (1) control (C), soil with no MP addition; (2) soil added with 0.2% (w/w) of PET (PET 0.2%); (3) soil added with 0.5% (w/w) of PET (PET 0.5%); (4) soil added with 0.2% (w/w) of PVC (PVC 0.2%); (5) soil added with 0.5% (w/w) of PVC (PVC 0.5%). A graphical representation of the experimental setup and the analyses described in the Materials and Methods section is provided in Figure 1.

A commercial soil with the following characteristics was used in this experiment: peaty‐clayey, pH (in H_2_O) 6.5, electrical conductivity 0.3 dS m^−1^, density 0.2 g cm^−3^, total porosity 47% v/v. Elemental concentrations in the soil were determined as in Bettarini et al. (2019) and single ion concentration was: 4.4 ± 0.2, 19.7 ± 0.5, 2.6 ± 0.1 mg g^−1^ d.w. for K, Ca, and Mg, respectively, and 205.2 ± 16.9, 69.2 ± 2.9, 38.5 ± 1.8, 15.0 ± 1.0, 113.2 ± 4.6, 315.4 ± 3.9, 6.3 ± 0.2 μg g^−1^ d.w. for Fe, Mn, Cu, Ni, Zn, Na, and Cd, respectively (Pb was below detection limit). Prior to the experiment, soil was oven‐dried at 60°C and sieved with a stainless‐steel sieve to obtain soil particles with a size ≤ 3 mm. Externally dark‐coated glass pots (7 cm diameter × 7 cm depth) were individually prepared by adding 20 g of dried sieved soil, with or without MPs, and water to field capacity. MPs were accurately mixed with dried soil inside glass containers in order to homogenise their distribution in the substrate. The above‐mentioned soil characteristics were checked after MP addition, and no significant differences were detected.

Seeds of *Arabidopsis thaliana* Col‐0 were stratified for 24 h in the dark at room temperature to promote synchronous germination. One seed per pot and 16 seeds per treatment were sown for a total of 80 plants. Plants were grown for 3 weeks in a climatic chamber with the following controlled conditions: 24/16°C day/night temperature, light intensity 300 μmol photons m^−2^ s^−1^, 16‐h (day) photoperiod, and relative humidity 60%–65%. The pots were watered weekly with 10 mL of distilled water to maintain soil field capacity, as shown by Pignattelli et al. (2020).

Plant growth was monitored by measuring the rosette area at different times (10, 14 and 21 days after germination) with a digital camera (Canon PowerShot SX100 IS) appropriately placed above the pots at a constant distance of 40 cm from the pot bottom. The images were analysed with the Fiji software ImageJ (Schindelin et al. 2012), and the rosette area was expressed in cm^2^. Rosette fresh and dry weights of a subset of plants (8 per treatment) were also determined. However, root growth was not analysed since secondary roots of *Arabidopsis* have a fine architecture, and plants could not be uprooted without substantial damage and loss of biomass.

### 2.2 Determination of Malondialdehyde Concentration

Malondialdehyde (MDA) concentration was determined after extraction in trichloroacetic acid (TCA), using three‐week‐old fully expanded leaves. MDA concentration was measured using the thiobarbituric acid (TBA) method (Heath and Packer 1968). For 1 mL of extract, 1 mL of 0.5% TBA in 20% TCA was added; the mixture was heated at 95°C for 30 min, cooled in an ice bath to end the reaction, and then centrifuged at 10.000 × g for 10 min. The supernatant absorbance was recorded at 532, 600, and 450 nm, and MDA concentration was calculated using the following formula: C (μmol L^−1^) = 6.45 (A_532_–A_600_)—0.56 A_450_. The concentration was then expressed in μmol g^−1^.

### 2.3 Analysis of Photosynthesis, Chlorophyll Content and Stomatal Features

Plant gas exchanges were measured on three‐week‐old fully expanded leaves of *A. thaliana* using a Li‐Cor 6400 XT portable photosynthesis system (Li‐Cor). Leaves were placed inside the 2‐cm^2^ leaf cuvette with the following settings: 400 ppm of CO_2_ concentration, 200 μmol m^−2^ s^−1^ of light intensity, and a leaf temperature of 22°C. Net photosynthesis (A_n_, μmol m^−2^ s^−1^) and stomatal conductance (g_s_, mmol m^−2^ s^−1^) were measured after reaching steady‐state conditions.

Chlorophyll *a* fluorescence measurements were performed on three‐week‐old fully expanded leaves using a portable fluorimeter (HandyPEA, Hansatech Instruments Ltd.). Briefly, the fluorescence emission was evaluated with the application of a 1 s‐long saturating light pulse (3500 μmol m^−2^ s^−1^, 650 nm) on 20‐min dark‐adapted leaves, between 10:00 and 11:00 am. The obtained increase of chlorophyll fluorescence (OJIP transients) was double normalised between *F*_*0*_ (step O, minimum fluorescence value at 20 μs from the light pulse) and *F*_*M*_ (plateau P, maximum fluorescence value at chlorophyll fluorescence plateau) to allow comparisons between treatments, and then analysed according to Stirbet and Govindjee (2011). Basic selected parameters were calculated with the Biolyzer software (Fluoromatic Software). Parameters considered in this study are listed and described in Table S2 according to Dainelli et al. (2024).

Chlorophyll content (ChlM) was measured from leaf transmission in the far red and near infrared (Cerovic et al. 2008; Gitelson et al. 1999) with a portable multi‐pigment metre (MPM‐100, Opti‐Sciences) on the same day and the same leaves used for chlorophyll *a* fluorescence measurements.

Stomatal density and size were assessed using the nail polish method (Pathoumthong et al. 2023; Wu and Zhao 2017). Briefly, the adaxial and abaxial surfaces of three‐week‐old leaves were covered with a thin layer of clear nail polish; clear tape was then used to remove the dried nail polish, obtaining the leaf imprints. Stomatal density (D) was determined by counting stomata on these leaf imprints using an inverted light microscope (Carl Zeiss Axio Vert.A1) in six different 1 mm^2^‐wide areas per leaf. Areas were selected on both sides (adaxial and abaxial) of the midrib and homogeneously distributed from the petiole to the leaf apex. To measure stomatal size, 10 stomata per imprint were randomly selected, and their guard cells were measured using the Axio Vert 3.1 software; stomatal size (S, μm^2^) was calculated as in Ouyang et al. (2017), multiplying length by width of the guard cells.

### 2.4 Determination of Macro‐ and Microelement Concentration in Arabidopsis Plants

Element concentrations in *A. thaliana* shoots were determined following the methodology employed by Bettarini et al. (2019) and Colzi et al. (2022). Plant rosette was carefully washed with demineralised water, oven‐dried to constant weight, and wet‐digested using 10 mL of 69% HNO_3_ in a microwave digestion system (Mars 6, CEM) with a maximum temperature of 200°C for 20 min. Macronutrient (K, Ca and Mg) and micronutrient (Zn, Mn, Fe and Cu) concentrations were determined by atomic absorption spectroscopy (PinAAcle 500, Perkin Elmer) and expressed on a dry weight basis. Certified reference materials (grade BCR, Community Bureau of Reference; Fluka Analytical, Sigma‐Aldrich) were used to verify the method's reliability and accuracy (values < 10% and < 5% RSD, respectively).

### 2.5 Botrytis Cinerea Inoculation and Disease Quantification

Leaves from three‐week‐old *A. thaliana* plants were detached and placed in Petri dishes containing, on the bottom, a filter paper disc wet with 1 mL of sterile water. A small layer of moistened paper was placed on the leaf petiole to cover it. Two leaves per plant, from a total of eight plants per condition, were inoculated with the fungal pathogen *Botrytis cinerea* strain B05.10 previously grown on potato dextrose agar (PDA for microbiology, VWR International srl, Italy) at 21/18°C (day)/(night), with 12 h of light (100 μmol m^−2^ s^−1^) to stimulate and standardise conidia production (Baccelli et al. 2022). Conidia were collected from mycelium after 10–15 days of growth and used to inoculate leaves. Leaves were inoculated by applying, next to the midrib, a single 5‐μL droplet of a conidial suspension (5 × 10^5^ conidia ml^−1^) in potato dextrose broth (PDB, Laboratorios Conda S.A., Spain). Petri dishes containing leaves were sealed with parafilm to maintain high relative humidity conditions needed for infection development and incubated at 20°C–23°C with 12 h of light (approximately 50 μmol m^−2^ s^−1^). Disease severity was determined after 48 h by measuring the necrotic lesion diameter with a digital calliper.

### 2.6 Phytoalexin Production Assay

In this study, we employed the cerato‐platanin protein (Uniprot ID P81702), a well‐characterised fungal protein with established PAMP activity, as a model molecule to simulate plant‐pathogen interactions. Notably, this protein, produced by the ascomycete *Ceratocystis platani*, has been reported to trigger the activation of the plant immune system in various species, including *Platanus acerifolia* and *A. thaliana* (Lombardi et al. 2013; Baccelli et al. 2014). The yeast *Pichia pastoris* was used as a heterologous system to produce cerato‐platanin, using a well‐established pipeline described by Pazzagli et al. (2009). The pPIC9‐cp plasmid enabled the direct recovery of the protein from the culture filtrate (60 mg L^−1^ of culture medium) with a single purification step using Reverse‐Phase High‐Performance Liquid Chromatography (RP‐HPLC). Both the biological activity and structure of pure recombinant cerato‐platanin were compared with those of the native protein (Luti et al. 2016). Phytoalexin production assay was performed according to Pazzagli et al. (2009) with small changes. Briefly, two leaves per plant, from eight plants per condition, were removed from each plant and placed directly on sterile 100 × 20 mm Petri dishes containing moist filter paper. On the lower surface of one leaf, 10 μL droplets containing 150 μM soluble cerato‐platanin were added, whereas the other leaf was added with water droplets. Petri dishes were sealed and incubated at 25°C in continuous light (100 μmol m^−2^ s^−1^). After 24 h, droplets were withdrawn, and each spot on the leaves was washed twice with 10 μL of water. This step was performed in order to completely recover phytoalexins from the leaf surface. The collected droplets, with a total volume of 30 μL per sample, were transferred to a quartz cuvette with dark walls. Fluorescence was then recorded in a fluorescence spectrometer (Cary Eclipse Fluorescence Spectrometer, Agilent) at λ_ex_ = 320 nm and λ_em_ = 386 nm, slit 5 nm. Phytoalexin production was expressed as arbitrary units of fluorescence per drop (U.A. per drop).

### 2.7 Reactive Oxygen Species (ROS) Detection After Cerato‐Platanin Treatment

ROS detection on plant leaves was performed as reported in Baccelli et al. (2022), with small modifications. Briefly, cerato‐platanin at a concentration of 150 μM or water as a negative control was applied as 10‐μL droplets on the abaxial surface of detached *A. thaliana* leaves. Two droplets of cerato‐platanin or water were applied to the left or right blades, and three leaves per treatment were analysed. Incubations were performed in Petri dishes for 30 and 60 min, and ROS production was visualised with the cell‐permeable probe 2,7‐dichlorodihydrofluorescein diacetate (H2DCFDA; Sigma‐Aldrich). For staining, leaves were soaked for 1 h in a 10 μM H2DCFDA solution in 20 mM phosphate buffer, pH 6.8. After H2DCFDA incubation, leaves were washed twice with phosphate buffer and mounted on glass slides for microscopy analysis, which was performed under a confocal Leica TCS SP5 scanning microscope (Leica; λ_ex_ 460 and λ_em_ 512 nm).

### 2.8 LC–MS/MS Untargeted Metabolomics

Three random plants per treatment, including control and PET/PVC at both concentrations, were selected for the analysis of specialised metabolites (SMs). Specifically, three fully expanded three‐week‐old leaves per plant were detached and treated with either H_2_O as control (i.e., no infection), *B. cinerea* conidia, or cerato‐platanin (please refer to Sections 2.5 and 2.6 for details). After 24 h, the leaves were weighed and immediately powdered in liquid nitrogen and lyophilised at −60°C in a Modulyo freeze‐dryer system. The metabolite extraction protocol was adapted from Routaboul et al. (2006). Briefly, 1.8 mL of extraction solution (methanol/water/acetone/trifluoroacetic acid, 30/42/28/0.05% V/V) and 500 ng of Apigenin (used as internal standard) were added to 4–5 mg of lyophilised powder. Each sample was homogenised using a FastPrep instrument (1 min, 13,000 g). The samples were placed in an ultrasonic bath (Advantage‐Lab, AL04‐04‐230) for 1 min at 25 kHz and 4°C, shaken for 30 min at 4°C using a ThermoMixer C (Eppendorf), and centrifuged for 10 min at the maximum speed to remove debris. The supernatants were transferred to new tubes, and the pellets were extracted again with 1.8 mL of extraction solution, ultra‐sonicated, shaken, and centrifuged as in the first step. The second supernatant was pooled with the first in a glass tube, dried in a SpeedVac rotary evaporator, and freeze dryer. The dry pellet was resuspended in 200 μL of ULC/MS grade water (Biosolve) with 10% acetonitrile ULC/MS grade (Biosolve) and filtered through a Whatman glass filter (grade GF/D, 2.7 μm pore size).

Metabolomic data were acquired using an HPLC system (Ultimate 3000 Thermo) coupled with a quadrupole time‐ofof‐flight mass spectrometer (Q‐Tof Impact II Bruker Daltonics). Chromatographic separation and data‐dependent acquisition (DDA) methods for mass spectrometer data in positive and negative electrospray ionisation (ESI+ and ESI‐) modes were carried out as in Boutet et al. (2022). The obtained data files and MS/MS data files were converted to mzXML format using the MSConvert software (ProteoWizard package 3.0; Chambers et al. 2012) and processed using MZmine 3.6.0 software (http://mzmine.github.io/); software parameters are described in detail in Data S2.

Metabolite annotation was carried out following the steps outlined by Boutet et al. (2022). To further refine the annotations and improve unclear ones, the machine learning tool MS2query (de Jonge et al. 2023) was also employed. A minimum Tanimoto score of 0.65 was set as the threshold for accurate identification. Molecular networking was realised using the MetGem software (Olivon et al. 2018; https://metgem.github.io) with a cosine score threshold of 0.65 for both ESI+ and ESI‐ datasets; Cytoscape software (Shannon et al. 2003; https://cytoscape.org/) was employed to format metabolic categories and visualise metabolite networks (i.e., clusters of highly structurally similar compounds based on MS/MS spectra), as in Boutet et al. (2022). Confidence levels ranging from 1 to 4 were assigned to each annotation: 1 represents exact matches with authentic standards, 2 denotes putatively annotated compounds verified through multiple sources, 3 corresponds to compounds identified as part of putative classes, and 4 indicates uncharacterised MS/MS signals (Sumner et al. 2007).

Metabolite accumulation was normalised according to the internal standard (Apigenin) and the weight of leaves used for the extraction. Accumulation was expressed in Relative units (RU), and features with constant accumulation across the samples were not included in subsequent statistical analysis.

### 2.9 Statistics and Data Visualisation

Concerning growth (i.e., rosette area, rosette fresh and dry weight), physiological parameters (i.e., peroxidation status, photosynthetic parameters and ionome), *B. cinerea* lesion diameter, and phytoalexin production after cerato‐platanin treatment, two‐way ANOVA was used to check the significance of differences (*p* < 0.05) among means of treatment groups. Polymer type and concentration were set as fixed factors using GraphPad Prism 8 (GraphPad Software). A HSD‐Tukey test was run for post hoc comparisons; the model was validated using Spearman's test and Shapiro–Wilk test to check for homoscedasticity and normality of the residuals, respectively.

The web server MetaboAnalyst version 6.0 (Pang et al. 2024; Xia et al. 2009) was used to process metabolite accumulation data (Data S3). The data were normalised by median, Log_10_ transformed, and scaled according to the server's default parameters (i.e., mean‐centred and divided by the standard deviation of each metabolite). The obtained dataset (Data S4) was used to perform Partial Least Square Discriminant Analysis (PLS‐DA) to differentiate between the experimental conditions. A separate PLS‐DA was conducted for each of the conditions described in Sections 2.5 and 2.6 (i.e., H_2_O, cerato‐platanin and *B. cinerea* ). Metabolic features (i.e., metabolites, hereafter MFs) with a Variable Importance in Projection (VIP) score greater than 1 in the first two components were selected for subsequent analysis, in accordance with previous studies (Akarachantachote et al. 2014; Zheng et al. 2023).

A Venn diagram was created with the jvenn tool (Bardou et al. 2014) to visualise the overlap of features affected by the different conditions. Principal component analysis (PCA) was conducted on the accumulation values of MFs with VIP > 1 using MetaboAnalyst version 6.0, and group differences were assessed for significance through PERMANOVA tests. The accumulation of these MFs was also visualised through heatmaps and clustering analysis performed in RStudio (R Core Team 2020) with the following packages: pheatmap (Kolde 2019), tidyverse (Wickham et al. 2019), and dendextend (Galili 2015). Pairwise *t*‐tests were then performed in MetaboAnalyst 6.0 between each microplastic treatment (i.e., PET/PVC 0.2/0.5%) and the control under each of the conditions mentioned above. Log_2_ fold changes (log_2_FC) were automatically calculated, and MFs were considered significantly induced or repressed compared to the control when the *p*‐value was < 0.05. GraphPad Prism 8 was used to generate histograms of induced/repressed features. Correlations (*r* > 0.9) among MFs with VIP scores > 1 were calculated based on their accumulation patterns, enabling the creation of co‐accumulation networks using the ‘ExpressionCorrelation’ app in Cytoscape software (Boutet et al. 2022). The shape and size of the MFs within the networks were determined based on the previously calculated log_2_FC from the pairwise comparison of PET 0.5% vs. C. From these networks, a selected set of annotated features from key metabolic categories was identified, and their patterns were illustrated using boxplots generated with GraphPad Prism 8. In this instance, accumulation values were normalised and expressed as a percentage in relation to the sample exhibiting the highest accumulation value (i.e., 100%), as in Boutet et al. (2022). The putative chemical structures of selected features were realised using the Simplified Molecular Input Line Entry System (SMILES) from PubChem (Kim et al. 2023) in the ChemDraw software version 22.2.0.

## 3 Results

### 3.1 Effects of Microplastics on Shoot Growth and MDA Concentration

**FIGURE 2:**
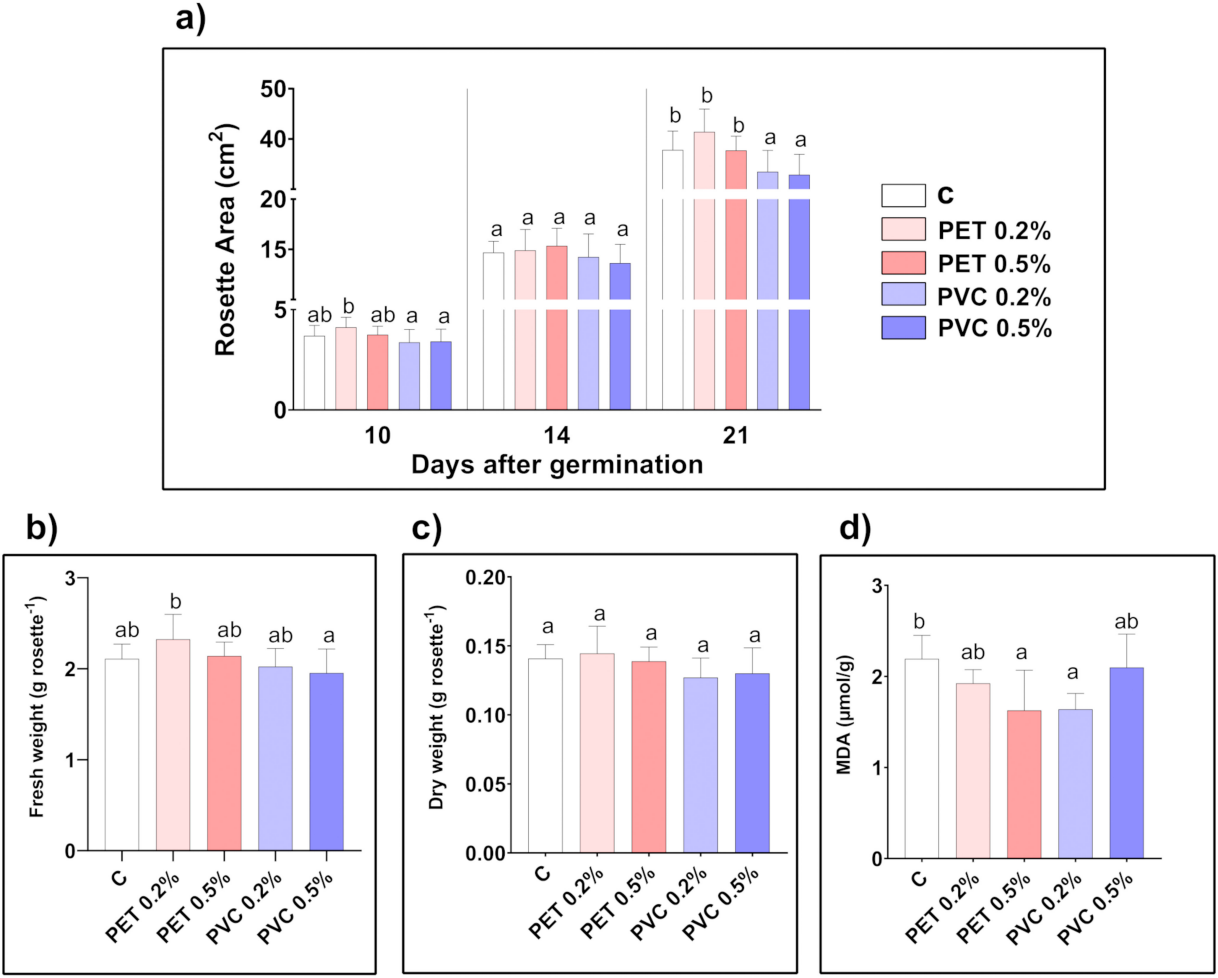
Growth and peroxidation status of *A. thaliana* plants after 21 days of growth in absence (C) or in presence of PET‐ or PVC‐MPs at different concentrations (0.2% and 0.5% w/w). (a) Rosette area (cm^2^) after 10, 14 and 21 days of growth (*n* = 16), (b) rosette fresh weight (g rosette^−1^; *n* = 8), (c) rosette dry weight (g rosette^−1^; *n* = 8) and (d) rosette MDA concentration (μmol g^−1^; *n* = 6). Values are mean of at least 6 replicates ± standard deviation. Lower case letters indicate significant differences among the samples (at least *p* < 0.05), at the same day of growth for rosette area and the end of the treatment for the other parameters.

PET‐MPs at both concentrations did not induce a significant change in *A. thaliana* rosette area compared to the control samples along the three sampling dates (Figure 2a). Conversely, PVC‐treated plants showed a reduced rosette area with respect to both control and PET‐treated plants, statistically significant 21 days after germination (−11% and −13% reduction in 0.2% and 0.5% PVC samples, respectively, compared to the control). Two‐way ANOVA highlighted a significant interaction of polymer type with concentration at this time point (*F* = 8.394 and *p* = 0.005).

Rosette fresh and dry weights were not affected by both MPs 3 weeks after germination (Figure 2b,c). Two‐way ANOVA did not highlight any significant result for the interaction of polymer type with concentration (*F* = 2.127, *p* = 0.1319 and *F* = 1.475, *p* = 0.2404 for fresh and dry weight, respectively).

MDA concentration measured 21 days after germination was significantly reduced (about 25%) in leaves of plants treated with PET 0.5% and PVC 0.2% in comparison with controls (Figure 2d). A significant interaction of polymer type with concentration resulted from two‐way ANOVA in this case (*F* = 5.099 and *p* = 0.0124).

### 3.2 Effects of Microplastics on Photosynthetic Parameters

Net photosynthesis (A_n_) was not significantly different among treatments, while stomatal conductance (g_s_) was significantly higher in PET‐treated plants, with an average increase of approximately 46% and 58% for the PET 0.2% and PET 0.5% concentrations, compared to the control (Table 1a). A significant interaction of polymer type with concentration was observed only for the g_s_ parameter in the two‐way ANOVA (*F* = 1.336, *p* = 0.2782 and *F* = 3.842, *p* = 0.0327 for A_n_ and g_s_, respectively).

**TABLE 1:** (a) Gas exchange parameters, (b) chlorophyll fluorescence parameters, (c) chlorophyll content and (d) stomatal density and size of *A. thaliana* plants grown for 3 weeks in absence (C) or in presence of PET‐ or PVC‐MPs at different concentrations (0.2% and 0.5% w/w).

a. Gas exchange parameters	Treatments				
	C	PET 0.2%	PET 0.5%	PVC 0.2%	PVC 0.5
A_n_	6.218 ± 0.638 a	6.437 ± 0.506 a	6.709 ± 0.721 a	6.375 ± 0.256 a	6.435 ± 0.627 a
g_s_	0.162 ± 0.032 a	0.236 ± 0.064 bc	0.255 ± 0.056 c	0.206 ± 0.031 abc	0.164 ± 0.009 ab

b. OJIP‐test parameters	Treatments				
	C	PET 0.2%	PET 0.5%	PVC 0.2%	PVC 0.5
F_0_	496.57 ± 10.37 ab	481.75 ± 14.67 a	493.29 ± 16.77 ab	511.00 ± 12.38 b	493.83 ± 6.11 ab
ΦPo	0.774 ± 0.005 ab	0.773 ± 0.005 ab	0.774 ± 0.008 b	0.764 ± 0.008 a	0.774 ± 0.006 b
ΨETo	0.590 ± 0.007 a	0.599 ± 0.017 a	0.596 ± 0.014 a	0.587 ± 0.007 a	0.583 ± 0.008 a
ΨREo	0.251 ± 0.021 a	0.288 ± 0.033 b	0.256 ± 0.035 ab	0.247 ± 0.009 a	0.225 ± 0.018 a
Sm	18.562 ± 1.242 a	21.131 ± 2.160 b	18.298 ± 1.989 a	17.926 ± 0.701 a	16.646 ± 0.962 a
ABS/RC	4.210 ± 0.167 a	4.301 ± 0.164 ab	4.283 ± 0.210 ab	4.484 ± 0.170 b	4.187 ± 0.209 a
Piabs	11.742 ± 0.844 a	11.901 ± 1.133 a	11.924 ± 1.660 a	10.293 ± 0.885 a	11.516 ± 1.097a

c. Chlorophyll content	Treatments				
	C	PET 0.2%	PET 0.5%	PVC 0.2%	PVC 0.5
ChlM	0.294 ± 0.040 a	0.296 ± 0.020 a	0.280 ± 0.015 a	0.267 ± 0.026 a	0.281 ± 0.033 a

d. Stomatal size and density	Treatments				
	C	PET 0.2%	PET 0.5%	PVC 0.2%	PVC 0.5
D_adaxial_	40 ± 7 a	47 ± 8 ab	46 ± 11 ab	55 ± 7 b	51 ± 11 ab
S_adaxial_	14.1 ± 2.2 a	16.0 ± 4.0 a	15.8 ± 3.6 a	15.2 ± 3.1 a	16.0 ± 1.4 a
D_abaxial_	126 ± 27 ab	135 ± 23 b	112 ± 25 ab	110 ± 20 ab	97 ± 17 a
S_abaxial_	18.6 ± 3.9 a	16.7 ± 3.0 a	16.5 ± 4.4 a	17.4 ± 2.2 a	16.3 ± 5.1 a

*Note:* Values are mean of 6 replicates ± standard deviation for gas exchange parameters and 8 replicates ± standard deviation for chlorophyll fluorescence parameters, pigment content and stomatal density and size. Letters indicate significant differences among the treatments (at least *p* < 0.05).

Concerning OJIP‐test parameters measured by chlorophyll *a* fluorescence (Table 1b), plants treated with PET 0.2% showed significantly higher mean values of ΨREo and Sm than controls (+15 and + 14%, respectively). Plants treated with PVC showed 0.2% higher ABS/RC (+7%) compared to controls. All other parameters were not significantly different across treatments. A significant interaction between the two fixed factors was verified by two‐way ANOVA only for *F*_0_ and Sm (*F* = 7.451, *p* = 0.0017 and *F* = 4.904, *p* = 0.0122 for *F*_0_ and Sm, respectively).

No statistically significant differences were observed in chlorophyll content across treatments (ChlM, Table 1c). Regarding stomatal features (density (D) and size (S) on the adaxial and abaxial leaf surface), plants treated with PVC 0.2% showed significantly higher D_adaxial_ compared to the control (+36%). However, no significant differences were found in D_abaxial_ across treatments (Table 1d). Two‐way ANOVA did not reveal a significant interaction between polymer type and concentration for these parameters. However, treatment concentration significantly affected stomatal density, if considered as a single source of variation (*F* = 6.338, *p* = 0.0039 and *F* = 3.769, *p* = 0.0312 for D_adaxial_ and D_abaxial_, respectively).

### 3.3 Effects of Microplastics on Shoot Elemental Composition

**FIGURE 3:**
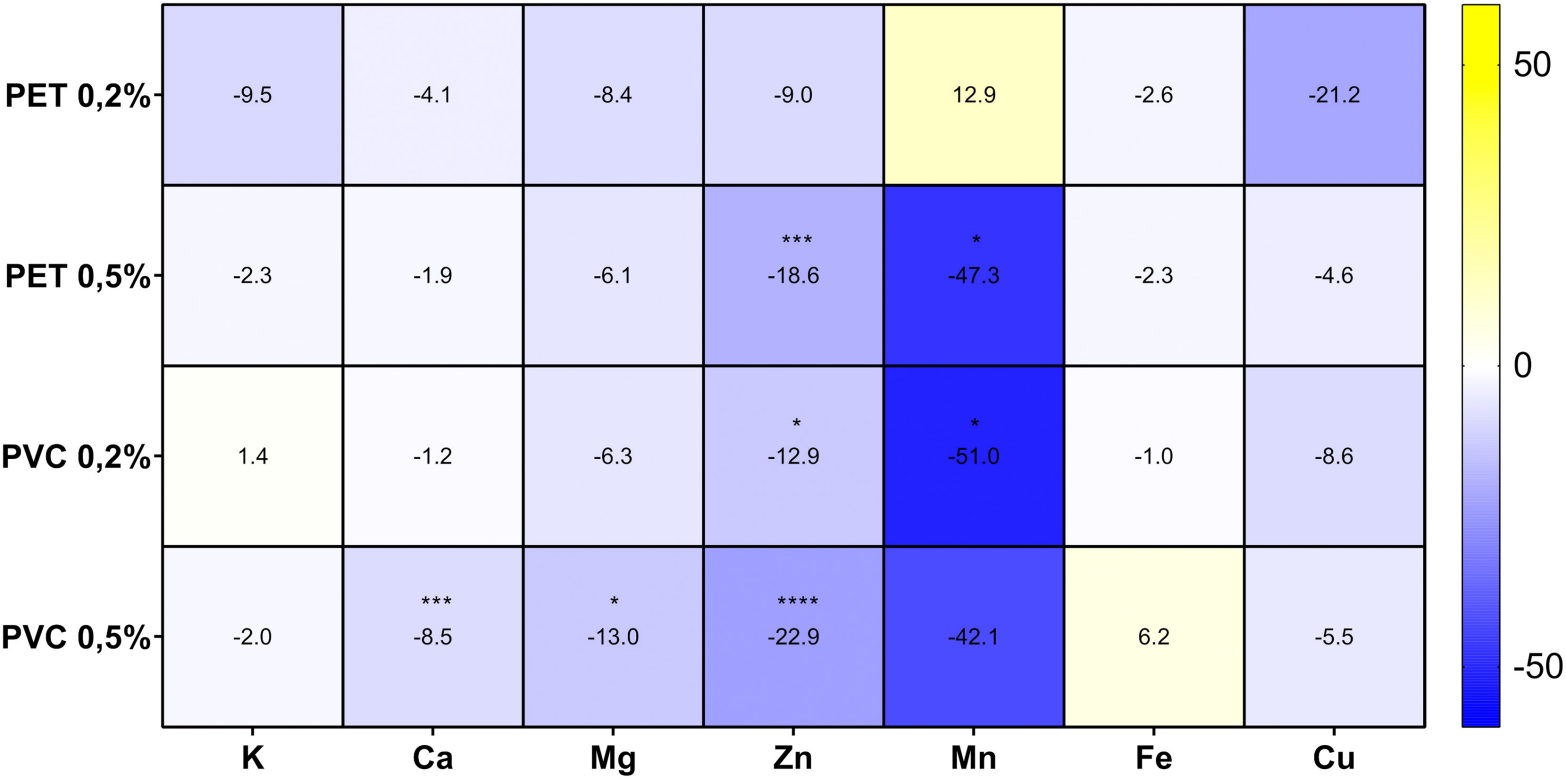
Heatmap showing ionome variation of shoots of *A. thaliana* plants grown for 3 weeks in absence (C) or in presence of PET‐ or PVC‐MPs at different concentrations (0.2% and 0.5% w/w). Colour scale indicates decreased (blue), unchanged (white) or increased (yellow) element concentration in respect to the control. The percentage of variation is reported, together with asterisks indicating the significant difference between the element concentrations in treated and control plants (at least *p* < 0.05).

The element concentrations in the shoots of plants 21 days after germination are reported in Table S3, while the amplitude of MP‐induced changes in ion levels than the control is shown with a heatmap (Figure 3). Concerning macronutrients, PVC 0.5% induced a significant reduction of Ca (−8.5%) and Mg (−13%) concentration compared to the control. Two‐way ANOVA highlighted a significant interaction polymer type × concentration for Ca, but not for Mg (*F* = 6.612, *p* = 0.0034 and *F* = 1.839, *p* = 0.1715 for Ca and Mg, respectively). No significant effect of the treatments on K shoot concentration was observed.

As for micronutrients, a reduction of Zn and Mn was observed in the shoots of all MP‐treated plants, except for PET 0.2% treatment. The micronutrient reduction was particularly heavy for Zn in PVC 0.5% plants (−22.9% than controls), and for Mn in PVC 0.2% plants (−51.0% with respect to controls). A significant interaction of polymer type with concentration was reported with two‐way ANOVA only for Mn, but not for Zn (*F* = 5.392, *p* = 0.0082 and *F* = 0.2709, *p* = 0.7640 for Mn and Zn, respectively). Regarding Fe and Cu concentrations, no significant differences were found among the treatments.

### 3.4 Effects of Microplastics on Plant Response to *Botrytis cinerea* or Cerato‐Platanin

**FIGURE 4:**
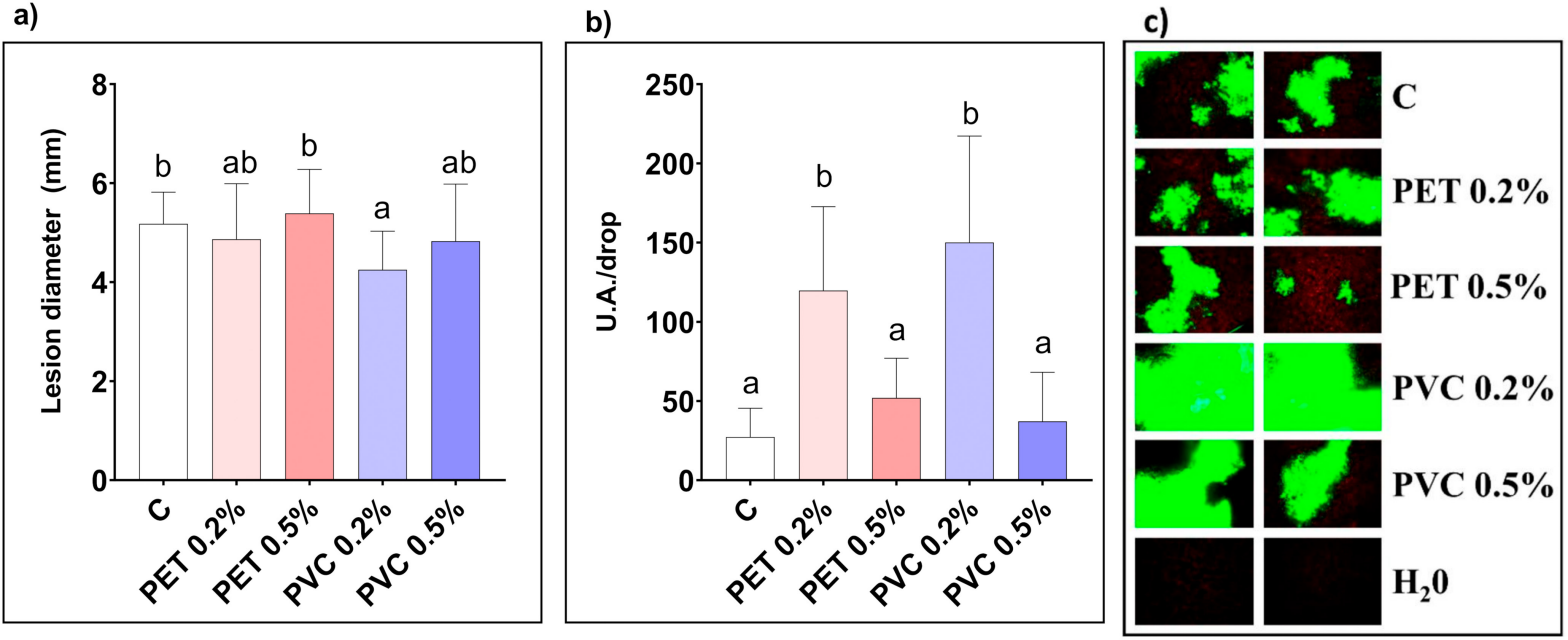
(a) Necrotic lesion diameter (mm) after 48 h from *B. cinerea* inoculation and (b) phytoalexin production (U.A. per drop) after 24 h of cerato‐platanin (CP) exposure in leaves of *A. thaliana* plants grown for 3 weeks in absence (C) or in presence of PET‐ or PVC‐MPs at different concentrations (0.2% and 0.5% w/w). Values are mean of 8 replicates ± standard deviation for phytoalexins and 24 replicates ± standard deviation for lesion diameter. Lower case letters indicate significant differences among the sample means (at least *p* < 0.05). (c) Confocal images showing ROS (in green) after 30 min of cerato‐platanin (CP) exposure in leaves of *A. thaliana* plants grown for 3 weeks in absence (C) or in presence of PET‐ or PVC‐MPs at different concentrations (0.2% and 0.5% w/w). Two representative leaves per treatment are shown; H_2_O indicates the negative control, that is absence of cerato‐platanin (CP) exposure.

After infection with the fungus *B. cinerea* , plants grown in the presence of PVC 0.2% showed a significantly lower lesion area (−18%) compared to the control (Figure 4a). No significant interaction of polymer type with concentration was found by two‐way ANOVA (*F* = 1.742 and *p* = 0.1791), even if the two sources of variation had a significant effect if considered individually (*F* = 6.921, *p* = 0.0095 and *F* = 6.883, *p* = 0.0014, respectively, for plastic type and concentration). Independent experiments yielded similar results, confirming that PVC at 0.2% concentration conferred protection to *A. thaliana* leaves against *B. cinerea* (Figure S1; Data S1).

Concerning the response of *A. thaliana* leaves to the PAMP protein cerato‐platanin, both PET and PVC treatment at the lowest concentration (0.2%) resulted in a significantly higher production of plant phytoalexins compared to the control (+338% and + 449% for PET 0.2% and PVC 0.2%, respectively, Figure 4b). Two‐way ANOVA did not highlight any significant interaction of polymer type with concentration (*F* = 1.334 and *p* = 0.2743) and, in this case, only the treatment concentration had a significant effect if considered as a single source of variation (*F* = 33.57 and *p* < 0.0001).

Cerato‐platanin induced ROS production in all conditions tested. The effect was particularly relevant in PVC 0.2% plants, and less evident in PET‐treated plants after a 30‐min incubation (Figure 4c). After 60 min of incubation, however, ROS decreased in all conditions (Figure S2).

### 3.5 Specialised Metabolite Profiles

#### 3.5.1 The Metabolomic Profile of *A. thaliana* Leaves Exposed to MPs and Biotic Stress

**FIGURE 5:**
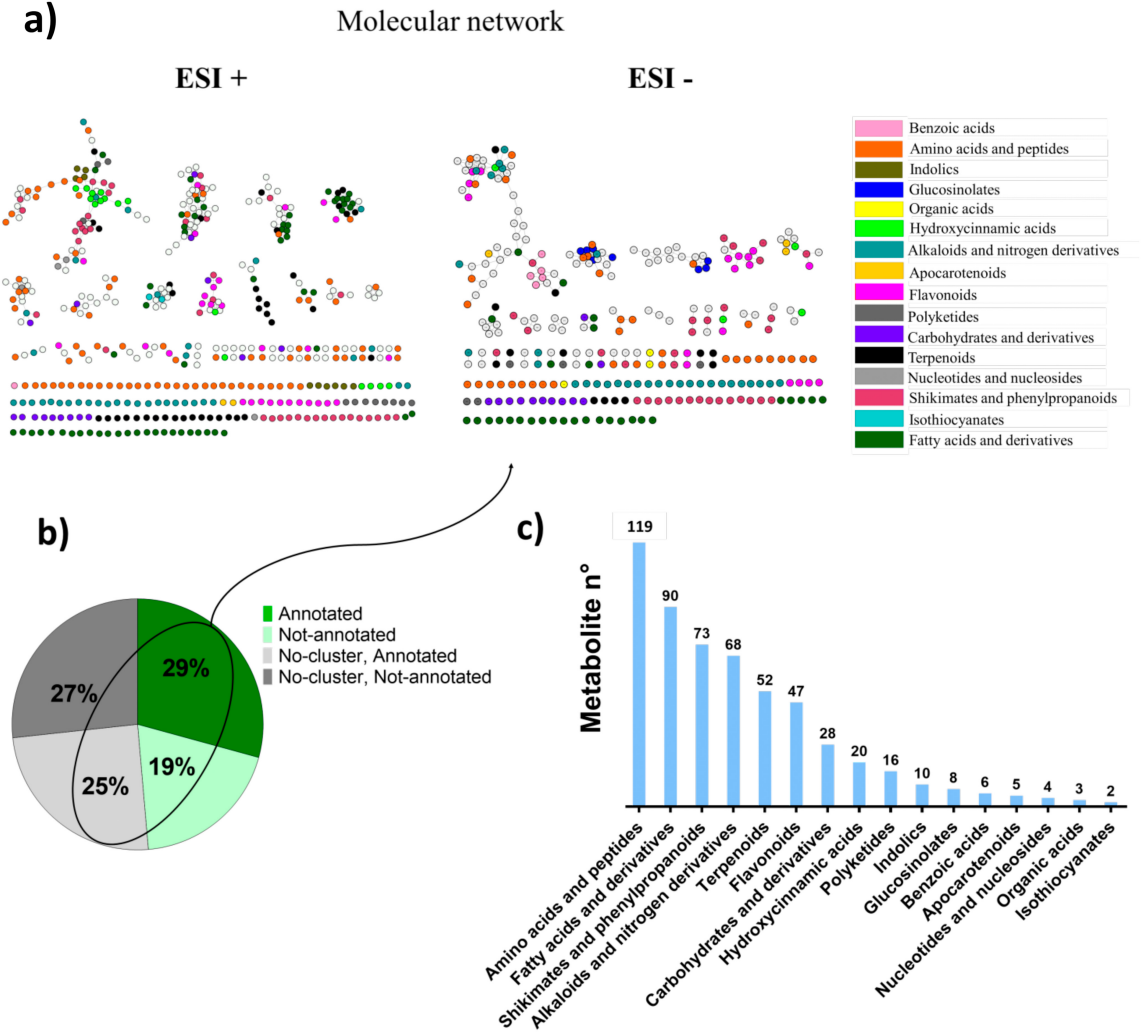
Metabolic diversity in *A. thaliana* leaves across all experimental conditions. (a) Molecular networks in ESI+ and ESI‐, realised with cosine similarity scores of 0.65 for both ionisation modes. Different colours represent distinct metabolic categories, as indicated in the legend. (b) Percentage of annotated MFs and their representation in the metabolic networks. (c) Histogram displaying the number of MFs per metabolic category, with values decreasing from left to right.

LC–MS/MS metabolomic analyses allowed the detection of 1022 metabolic features (MFs): 585 in ESI+ mode and 437 in ESI‐ mode. Metabolite annotation relied on different strategies, including molecular networking (Figure 5a), which facilitated the assignment of a large number of MFs to metabolic categories (Figure 5b) in *A. thaliana* leaves, with 185 metabolites hypothesised based on spectral library matching (Data S5). Several metabolic categories were identified (Figure 5c), the top five being: amino acids and peptides (119 MFs), fatty acids and derivatives (90 MFs), shikimates and phenylpropanoids (73 MFs), alkaloids and nitrogen derivatives (68 MFs) and terpenoids (52 MFs). Normalised intensity of all MFs was used to perform PLS‐DA under the three different applied conditions (i.e., H_2_O as control, cerato‐platanin and *B. cinerea* ), as described in Section 2.9. VIP scores for the first two components of each PLS‐DA and each MFs are reported in Data S6. MFs with VIP > 1 in both the first two components were those that best discriminated between treatments in the PLS‐DA analysis. These features, differentially accumulated across growth conditions (i.e., without MPs or with PET/PVC at 0.2% and 0.5%), were more abundant after *B. cinerea* infection (*n* = 403), followed by the other two conditions in decreasing order: H_2_O (*n* = 346) and cerato‐platanin (*n* = 81). Most of the selected metabolites were specific to a single condition (Figure S3, Data S1), accounting for approximately 71% of the total. These MFs were used for further statistical analysis.

#### 3.5.2 Effects of Microplastics on Plant Specialised Metabolism

**FIGURE 6:**
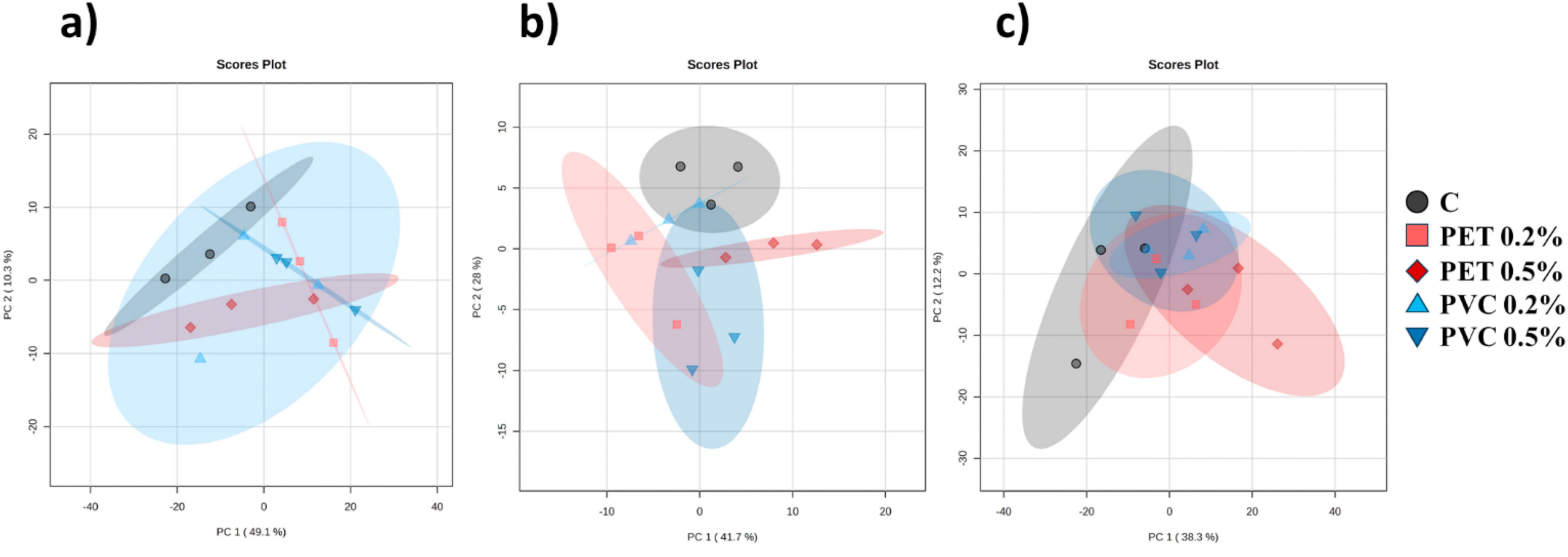
Principal Component Analysis (PCA) obtained by the analysis of MFs with VIP > 1 in the three different conditions: H_2_O as control (*a*), cerato‐platanin (CP) elicitation (*b*) and *B. cinerea* infection (*c*). Different colours, as indicated in the legend, represent different experimental growth conditions, specifically *A. thaliana* plants grown for 3 weeks in absence (C) or in presence of PET‐ or PVC‐MPs at different concentrations (0.2% and 0.5% w/w). Additionally, 95% confidence ellipses are also shown.

The three PCAs generated from the analysis of MFs selected by PLS‐DA with VIP score > 1 are shown in Figure 6. The percentage of variation explained by the first two components (PC1 and PC2) ranges from a maximum of 69.7% under the cerato‐platanin condition to a minimum of 50.5% under the *B. cinerea* condition. Although the five treatment groups (see Section 2.1) and their confidence ellipses appear to overlap considerably with each other, especially in H_2_O and *B. cinerea* conditions, PERMANOVA revealed statistically significant differences between them following cerato‐platanin elicitation and fungal infection (*F* = 6.941, *p* = 0.001 and *F* = 3.773, *p* = 0.007 for cerato‐platanin and *B. cinerea* , respectively). In contrast, no significant differences were reported after H_2_O exposure (control; *F* = 1.898 and *p* = 0.159). Pairwise PERMANOVA results are reported in Table S4. No significant differences were found, despite high F and R^2^ values, low P‐values, and distinct spatial distribution in the PCAs, especially in the comparisons between the control and the treatments (notably C vs. PET 0.5%).

**FIGURE 7:**
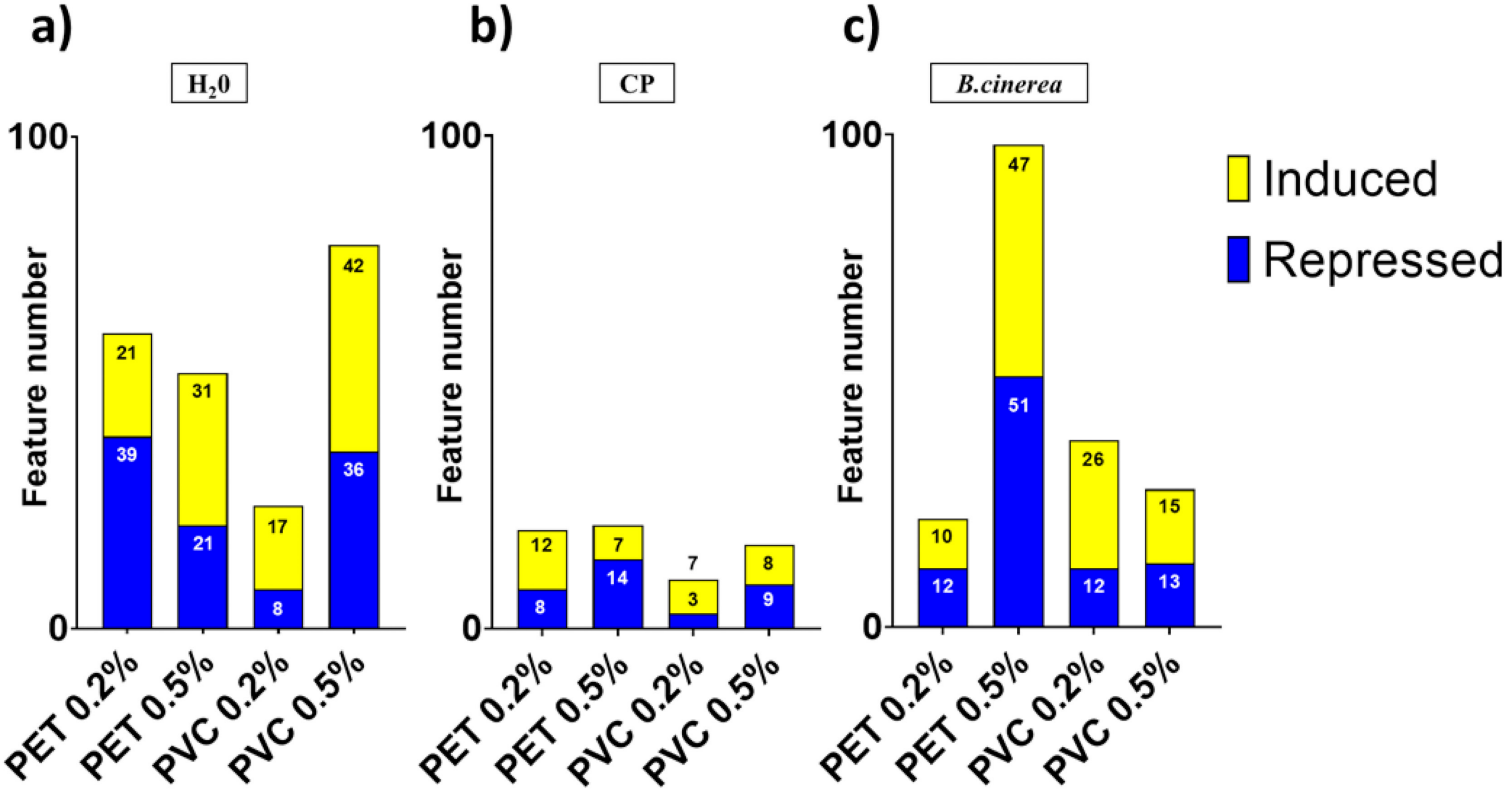
Histograms showing the number of significantly (P‐value < 0.05) induced (blue) and repressed (yellow) MFs from pairwise *t*‐test comparisons between each microplastic treatment (i.e., PET and PVC at 0.2% and 0.5%) and the control across the three different conditions: H_2_O as control (a), cerato‐platanin (CP) elicitation (b) and *B. cinerea* infection (c). The exact number of MFs is also indicated within the histograms.

Heatmaps in Figure S4 illustrate the accumulation trends of MFs showing differential accumulation among conditions and selected by PLS‐DA according to a VIP score > 1. Pairwise *t*‐tests between each MP treatment and the control under each condition were performed to assess the statistical significance of MF changes, and log_2_FC (treatment/control) values were calculated. Figure 7 presents histograms illustrating the number of significantly (*p*‐value < 0.05) induced and repressed MFs for each treatment compared to the control (untreated) condition. The corresponding *p*‐values, along with *t*‐stat and log_2_FC values for these differentially accumulated MFs (DAMFs hereafter) are reported in Data S7. The highest number of DAMFs was found in the pairwise comparison PET 0.5% vs. C during *B. cinerea* infection (*n* = 98, with 51 repressed and 47 induced), while the second highest was reported for the comparison PVC 0.5% vs. C in control conditions, that is, H_2_O (*n* = 78, with 36 repressed and 42 induced). In PVC vs. C, a greater proportion of DAMFs were induced rather than repressed, while in PET vs. C, repressed DAMFs generally exceeded the induced ones. A lower number of DAMFs was observed in cerato‐platanin‐treated leaves across all MP treatments.

Co‐accumulation networks (Figure S5), constructed solely for the H_2_O (untreated) and *B. cinerea* conditions due to their showing the highest number of DAMFs (Figure 7), revealed distinct clusters of metabolites showing similar accumulation behaviour (*r* > 0.9) in response to MP treatments. Among the identified modules in the H_2_O condition (Figure S5a), some were particularly enriched in flavonoids, shikimates, and phenylpropanoids, and hydroxycinnamic acids. Similar patterns, though less pronounced, were also observed under the *B. cinerea* condition (Figure S5b). Following the fungal attack, isothiocyanates exhibited greater abundance and larger sizes (i.e., higher log2FC values than the control).

**FIGURE 8:**
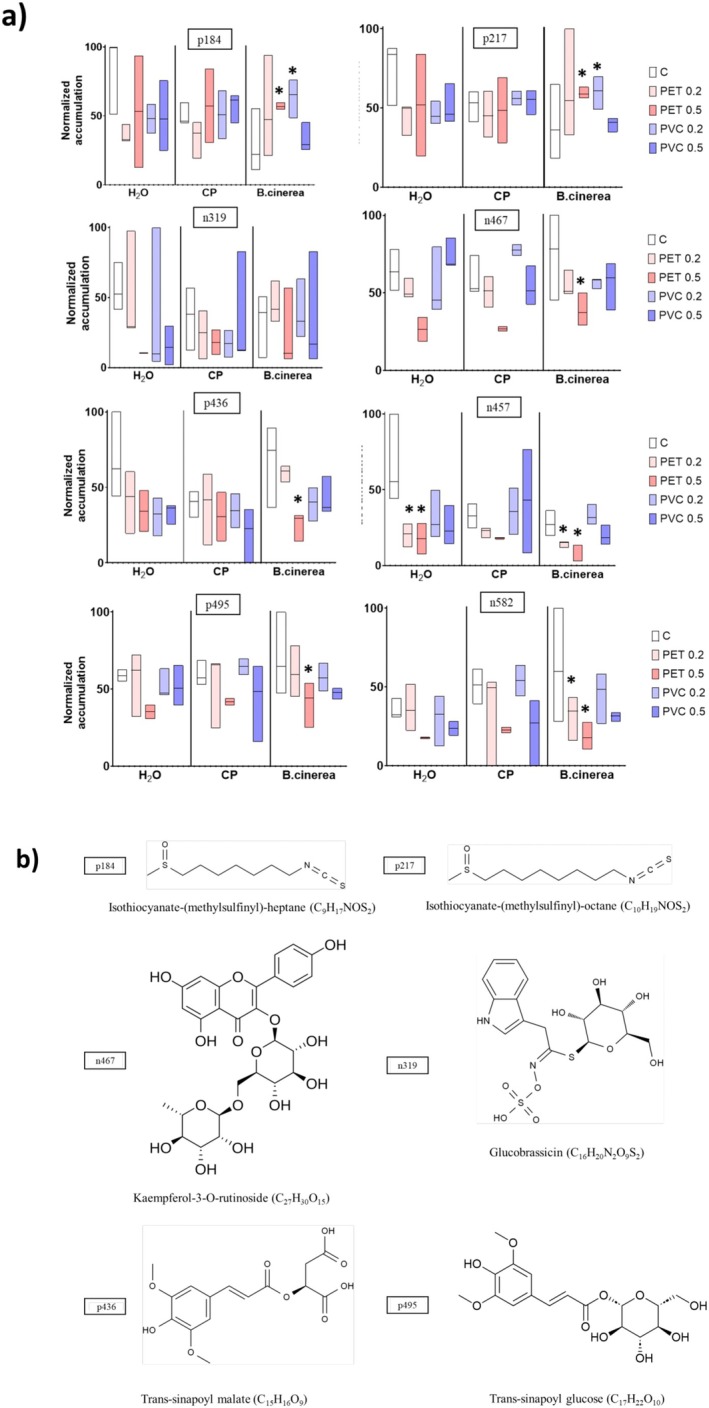
(a) Boxplots of selected DAMFs across all experimental conditions. Accumulation values (3 replicates per experimental condition) were normalised and expressed as a percentage in relation to the sample exhibiting the highest accumulation value (i.e., 100%). Asterisks on the boxplots indicate significance of pairwise *t*‐test between MP treatments and the control in the specific condition (i.e., H_2_O as control, CP elicitation and *B.cinerea* infection). (b) Putative chemical structures and molecular formulas of annotated DAMFs.

Figure 8a,b present the accumulation patterns and putative chemical structures for a subset of DAMFs (Section 2.9) associated with the specific metabolic categories mentioned earlier; Table 2 shows their annotation confidence level, significant pairwise *t*‐test results, and log_2_ FC values. Regarding isothiocyanates (p184 and p217), their accumulation significantly increased in leaves from PET 0.5% and PVC 0.2% plants compared to the control following *B. cinerea* infection. DAMFs p184 and p217 were annotated as isothiocyanate‐(methylsulfinyl)‐heptane and isothiocyanate‐(methylsulfinyl)‐octane, respectively. Conversely, an opposite trend was noted under H_2_O condition, with the highest accumulation values for the aforementioned DAMFs found in the leaves of plants grown in MP‐free substrates. No differences in the accumulation of the identified glucosinolate glucobrassicin (n319) were observed between the experimental conditions.

**TABLE 2:** Feature IDs and annotations of selected DAMFs shown in Figure 7.

ID	Metabolic category and annotation	Confidence level	Pairwise *t*‐tests with *p*‐value < 0.05; log_2_FC
p184	*Isothiocyanates* Isothiocyanate‐(methylsulfinyl)‐heptane	2	*B.cinerea*_PET 0.5% vs. C; Log2FC = 1.376 *B.cinerea*_PVC 0.2% vs. C; Log2FC = 1.389
p217	*Isothiocyanates* Isothiocyanate‐(methylsulfinyl)‐octane	2	*B.cinerea*_PET 0.5% vs. C; Log2FC = 0.943 *B.cinerea*_PVC 0.2% vs. C; Log2FC = 1.813
n319	*Glucosinolates* Glucobrassicin	1	N/A
n467	*Flavonoids* Kaempferol‐3‐O‐rutinoside	1	*B.cinerea*_PET 0.5% vs. C; Log2FC = −0.726
p436	*Shikimates and phenylpropanoids* (Hypothesis: trans‐sinapoyl malate)	3	*B.cinerea*_PET 0.5% vs. C; Log2FC = −1.251
n457	*Shikimates and phenylpropanoids*	3	H_2_O_PET 0.2% vs. C; Log2FC = −1.010 H_2_O_PET 0.5% vs. C; Log2FC = −1.029 *B.cinerea*_PET 0.2% vs. C; Log2FC = −0.897 *B.cinerea*_PET 0.5% vs. C; Log2FC = −1.452
p495	*Shikimates and phenylpropanoids* (Hypothesis: trans‐sinapoyl glucose)	3	*B.cinerea*_PET 0.5% vs. C; Log2FC = −0.612
n582	*Shikimates and phenylpropanoids*	3	*B.cinerea*_PET 0.2% vs. C; Log2FC = −0.937 *B.cinerea*_PET 0.5% vs. C; Log2FC = −1.497

*Note:* Annotation confidence levels are shown along with significant pairwise *t*‐test results and log_2_FC values.

One flavonoid (n467) and four shikimates and phenylpropanoids (p436, n457, p495, and n582) DAMFs were repressed in PET‐treated plants compared to the control, particularly at 0.5% concentration. DAMF n467 was annotated as a kaempferol‐3‐O‐rutinoside, whereas DAMFs p436 and p495 were annotated as trans‐sinapoyl malate and trans‐sinapoyl glucose, respectively. In contrast, PVC‐treated plants, particularly at a concentration of 0.2%, exhibited a higher accumulation of these DAMFs, more closely resembling the control values. Similar to isothiocyanates, control plants displayed the highest accumulation values for p436 and n457.

Normalised accumulations and the chemical structure of the phytoalexin camalexin (p139) are also presented in Figure S6 a,b. The results obtained under cerato‐platanin condition are consistent with those obtained via fluorescence spectroscopy (Sections 2.6 and 3.4). The same information is shown in Figure S6c–f for the annotated antioxidants (i.e., oxidised glutathione and ascorbic acid). No significant differences were observed in their accumulation across experimental conditions, although PVC‐treated plants showed higher mean values for the normalised accumulation of oxidised glutathione compared to the other treatments under cerato‐platanin conditions.

## 4 Discussion

### 4.1 Microplastics Altered Plant Physiological Status

Consequences of exposure and growth of plants with MPs are variable, and often negligible (see for example Wang, Luo et al. 2023; Jiang et al. 2024 and references therein). In this study, a 3‐week growth on PVC MPs moderately inhibited *A. thaliana* development (−10% reduction of shoot area). On the other hand, growth on PET MPs did not have any developmental effect on our plants, probably due to the well‐known low toxicity of PET (Chen et al. 2022a, 2022b; Colzi et al. 2022; Dainelli et al. 2023). Given the relatively large size (40–50 μm) of the MPs employed in this study, these particles may not infiltrate plant tissues, unlike their submicrometric counterparts (Li et al. 2020), and are not expected to undergo significant degradation within the three‐week period (Lin et al. 2022). Thus, the observed effects are probably due to a multifaceted and still to be fully elucidated mechanism that may involve alterations in soil structure, adsorption processes, and mechanical damage to the roots, rather than direct interference with the plant's internal physiological processes. In the case of polymers such as PVC, which may release radical species and dangerous by‐products (Rånby et al. 1978; Yuan et al. 2014), this could account for the moderately negative effects observed.

Plastic particles, and especially PVC, are generally reported to induce oxidative stress, increasing MDA concentration and decreasing plant biomass production (Pignattelli et al. 2020). However, we found a reduced level of lipid peroxidation in PET 0.5% and PVC 0.2% plants, indicating that MPs caused a mild oxidative stress, unable to largely impact plant growth, but perhaps sufficient to induce an antioxidant response, in turn lowering lipid peroxidation. Similar conditions have been reported for other oxidant pollutants when applied in low doses (Muszyńska and Labudda 2019). Differently, the antioxidant response could have counterbalanced a more intense oxidative stress induced by the higher concentration of PVC, resulting in no observable effects on MDA levels.

Photosynthesis was also unaffected by growth with MPs. Ameliorative effects of MPs on the photosynthetic activity have been sporadically reported, but the underlying mechanism remains unclear (Khan et al. 2024; Sun et al. 2024). Our photochemical data suggest that plants grown with PVC 0.2% could have a larger antenna size of PSII (as indicated by ABS/RC), while plants grown with PET 0.2% could have more efficient electron transport both because of the greater presence of carriers in the electron transport chain and because of a larger pool of electron acceptors at PSI (as indicated by SM and ΨREo, respectively). However, none of the observed changes was able to influence net photosynthesis at the leaf level. Chlorophyll content also remained constant among the treatments.

An increase in stomatal conductance was observed in plants grown under both 0.2% and 0.5% PET. This phenomenon was not determined by increased stomatal size or density, which was only observed in the adaxial side of plants treated with PVC 0.2% and did not affect photosynthesis. We speculated that increased stomatal conductance represents a side effect of PET‐imposed changes in soil structure. In fact, MPs have been reported to affect soil properties, with PET decreasing soil density and increasing soil water availability (de Souza Machado et al. 2019).

The analysis of plant elemental profile revealed that PET and PVC reduced the accumulation of some nutrients, most likely due to an impairment of the root functioning, as previously reported for such plastic polymers (Colzi et al. 2022; Dainelli et al. 2023). However, only the Mn concentration of PVC‐grown plants was under the optimum range for plant shoot growth (Marschner 1995; Kabata‐Pendias 2000), which might be associated with the reduced rosette development observed in PVC‐grown plants.

### 4.2 Microplastics Altered Plant Response to Biotic Stress Through a Potential Priming Effect

MPs and plant pathogens are pervasive in both natural and agricultural soils (Surendran et al. 2023), and their combined impact on plants is realistic. PVC 0.2%‐treated plants displayed a reduced diameter of *B. cinerea* lesions, suggesting decreased susceptibility to the pathogen attack. Furthermore, cerato‐platanin‐elicited plants grown in low concentrations (0.2%) of PET or PVC showed increased phytoalexin and ROS production, indicating enhanced responsiveness of the plant immune system. Phytoalexins may contribute to improving the resistance to *B. cinerea* . In particular, the *Arabidopsis* phytoalexin camalexin (Glawischnig 2007) plays an active antimicrobial role against *B. cinerea* (Kliebenstein et al. 2005). These results therefore suggest a priming effect of PVC 0.2% on *A. thaliana* plants (i.e., a condition of stronger and enhanced reactivity to stressors; Conrath et al. 2015), with low concentrations of MPs likely functioning as triggering stimuli, similarly to what has been shown for other different nanomaterials (González Guzmán et al. 2022).

The signalling pathways involved in plant responses to biotic stress (Anjali et al. 2023) are also reported to be activated in plants grown with MPs (Cui et al. 2024; Wang, Bai, et al. 2023; Wang, Xiang et al. 2023). ROS act as crucial signalling compounds triggering downstream processes and transcriptional regulation (Tyagi et al. 2022), including camalexin biosynthesis (Glawischnig 2007; Nguyen et al. 2022). Consequently, the stronger (30 min) ROS surge observed following cerato‐platanin elicitation suggests a central role for these molecules in the priming response activated by PVC 0.2%. ROS production could have been mediated by respiratory burst oxidase homologues (RBOHs), which use NADPH to reduce O_2_ (Wu et al. 2023) and generate ROS, important in plant immunity and abiotic stress mitigation (Chen and Yang 2019) through the regulation of specialised metabolite biosynthesis (Begum et al. 2024). PVC‐induced oxidative stress at 0.2% might have triggered an over‐reduced state, suggested by the low lipid peroxidation, thus providing more reducing power for RBOH activity and the subsequent strong ROS spike after cerato‐platanin elicitation.

Priming effects were not observed when MPs were employed at higher concentrations (0.5%). It is possible that high MPs exceed the optimal dose for ROS activation and the consequent synthesis of phytoalexins. In the case of PET 0.5%, the higher stomatal conductance might have contributed to counteract the positive effect of priming, as pathogens like *B. cinerea* are known to enter plants through active penetration, as well as natural discontinuities, including stomata (Müller et al. 2024). Due to the possible MP influence on stomatal conductance, dynamic changes to adequately regulate their opening could be expected in the complex signalling network of plant‐pathogen interaction, reported to involve also stomatal status and the stomatal regulator abscisic acid (AbuQamar et al. 2017; Gogoi et al. 2024).

### 4.3 Microplastics Altered Leaf Specialised Metabolism Influencing the Response to Biotic Stress

Brassicaceae such as *A. thaliana* possess a large diversity of specialised metabolic pathways (Barreda et al., 2025a, 2025b), as confirmed by the metabolomic profile presented here and in other studies (Wu et al. 2018). The presence of PET and PVC in the growth substrate induced various polymer‐ and concentration‐dependent changes in the specialised metabolome of *A. thaliana* , involving a variety of metabolic pathways that reflected a scenario as multifaceted as the MP impact on plant is, as mentioned in Section 4.1. Such changes can be associated with both the MP‐imposed damage and the MP‐induced plant response, consistently with previous studies on other species (e.g., Wang,Bai, et al. 2023; Xiao et al. 2024).

Specialised metabolites are also essential in plant responses to many pathogens, including *B. cinerea* (Anjali et al. 2023; Wu et al. 2023). The vast majority (447 out of 631) of the considered subset of MFs is unique to a specific condition, confirming that stress combination is perceived by plants as a novel stress state, with responses primarily influenced by the more severe and dominant stressor (Pandey et al. 2015). In our case, the biotic stress appears as the dominant stressor when examining the relevant MFs that overlap between the H_2_O condition and the fungal infection. Nevertheless, the presence of shared pathways is highlighted, as evidenced by the total number of shared MFs across experimental conditions (169 shared by any two conditions and 15 shared by all three), which belong to various metabolic categories (e.g., amino acids and derivatives, flavonoids, hydroxycinnamic acids, shikimates and phenylpropanoids, terpenoids, etc.). The observed lower number of glucosinolates, hydroxycinnamic acids, flavonoids, shikimates and phenylpropanoids, and terpenoids exhibiting differential accumulation across MP treatments after cerato‐platanin elicitation compared to H_2_O and *B. cinerea* conditions is likely due to the ability of this PAMP to induce local and specific immune pathways (Baccelli et al. 2014), thereby reducing the number of affected key metabolites. This could also account for the camalexin accumulation pattern, which is far more pronounced under cerato‐platanin treatment.

The significant results from the PCA analysis and subsequent PERMANOVA, observed exclusively under cerato‐platanin and *B. cinerea* conditions, emphasise that the differential effects of polymer type and concentration on specialised metabolism are intensified by the application of a combined stress. The larger separation along the first PCA component (explaining 38.3% of variance) and the higher number of DAMFs indicated that plants treated with the highest concentration of PET displayed the most distinct regulation of specialised metabolism compared to control plants in response to *B. cinerea* infection.

Interestingly, resistance to fungal attack was observed exclusively in PVC 0.2%‐treated plants, which also exhibited the lowest overall number of DAMFs when considering all conditions together (i.e., H_2_O, cerato‐platanin, and *B. cinerea* ). This suggests a mild modulation of specialised metabolism, which could underlie the priming mechanism. Notably, induced DAMFs exceeded the repressed ones by more than two times in PVC 0.2% plants after fungal infection. In contrast, the increased repression of specialised metabolites observed in the other treatments, and particularly with PET 0.5%, might be linked to several mechanisms, including negative regulation of gene expression through DNA methylation (Gallego‐Bartolomé 2020; Han et al. 2024; Marfil et al. 2019).

Among the recent studies about how plastic pollution influences plant metabolic responses, Wang, Bai, et al. (2023) proposed that specialised metabolites enhanced the resistance of *Spirodela polyrhiza* to PVC‐MPs water pollution. Whereas, Shi et al. (2024) demonstrated that low concentrations of polyethylene modulated the accumulation of flavonoids and cinnamic acid antioxidants in tomatoes subsequently exposed to herbivores. In our study, various flavonoids (e.g., kaempferol‐3‐O‐rutinoside) were repressed by the PET 0.5% treatment compared to the control. This effect aligns with findings by Wang, Bai, et al. (2023) in *S. polyrhiza* , even if using high doses of PVC‐MPs in hydroponics. Given that flavonoids are known antioxidants (Agati et al. 2012), and considering the minimal differences in other annotated antioxidants (e.g., glutathione and ascorbic acid), we hypothesise that additional ROS scavenging systems (e.g., α‐tocopherol etc.) and increased activity of scavenging enzymes may help reduce lipid peroxidation in plants grown with MPs, and in particular when using PET 0.5%.

Concerning defensive glucosinolate compounds, high doses of MPs are reported to decrease their production in Brassicaceae (López et al. 2022; Tong et al. 2024). In our experiment, where a more realistic low dose of MPs was tested, only minimal changes in glucosinolate levels were observed. However, a marked increase in certain annotated glucosinolate degradation products, such as putative isothiocyanate‐(methylsulfinyl)‐heptane and isothiocyanate‐(methylsulfinyl)‐octane, was notably detected in PVC 0.2% plants infected with *B. cinerea* . MPs have been shown to enhance myrosinase activity (Tong et al. 2024), the enzyme responsible for glucosinolate hydrolysis (Chhajed et al. 2020). The increased accumulation of these isothiocyanates (glucosinolates' degradation products) may therefore be related to a stimulation of myrosinase activity in PVC 0.2% plants, a response reported for other priming agents (Cachapa et al. 2021; Qing et al. 2023) and contributing to the hypothesis of a primed state in our samples. Isothiocyanates are indeed characterised by high biological activity and, together with other glucosinolate degradation products such as nitriles, are toxic for a wide range of plant and human pathogens (Hoch et al. 2024; Nowicki et al. 2019; Plaszkó et al. 2021).

Given the fungi toxic properties of isothiocyanates (Dubey et al. 2021), their increased accumulation might also explain the reduced development of *B. cinerea* lesions observed in PVC 0.2% plants. However, a similar accumulation of these molecules was observed in PET 0.5% plants, which did not exhibit a higher pathogen resistance. The accumulation of other shikimates and phenylpropanoids, specifically sinapate esters, was impaired in PET 0.5% plants following fungal infection. This impairment might have negatively influenced plant‐pathogen interaction, offsetting the benefits of isothiocyanates accumulation. Reduced levels of sinapate esters, in fact, have been linked to increased susceptibility of *A. thaliana* to fungal pathogens (König et al. 2014). Conversely, the observed increased resistance of PVC 0.2% plants to *B. cinerea* may result from a specialised metabolism profile that includes both elevated levels of isothiocyanates and stable concentrations of phenylpropanoids, such as trans‐sinapoyl malate and trans‐sinapoyl glucose.

## 5 Conclusions

This study has investigated possible interactions between MPs and pathogen stress in plants. We report mild effects on plant physiology of MPs at low (realistic) doses, but specific polymer‐concentration combinations may carry a significant priming effect, activating defensive responses against fungal pathogens. Fine regulation of these effects by specialised metabolites is also likely, and some possible metabolic interactions were highlighted. Many other factors can play a role in influencing the ecological and agronomic implications of exposure to MPs. For example, domestication can diminish both constitutive and inducible defences (Córdova‐Campos et al. 2012; Moreira et al. 2018; Soltis et al. 2018), and this may change the capacity to cope with biotic stress in crop species, even under an MP‐induced priming.

## Author Contributions

**Marco Dainelli:** writing – review and editing, writing original draft, methodology, investigation, formal analysis, data curation, conceptualization. **Costanza Cicchi:** writing – review and editing, methodology, formal analysis. **Ivan Baccelli:** writing – review and editing, methodology, formal analysis. **Stéphanie Boutet:** writing – review and editing, methodology, formal analysis. **Ilaria Colzi:** writing – review and editing, methodology, formal analysis. **Andrea Coppi:** writing – review and editing, methodology, formal analysis. **Simone Luti:** writing – review and editing, methodology, formal analysis. **Sara Pignattelli:** writing – review and editing, methodology, formal analysis. **Susanna Pollastri:** writing – review and editing, methodology, formal analysis. **Francesco Loreto:** writing – review and editing. **Luigia Pazzagli:** writing – review and editing. **Massimiliano Corso:** writing – review and editing, methodology, investigation, formal analysis, data curation. **Cristina Gonnelli:** writing – review and editing, writing original draft, resources, project administration, methodology, investigation, data curation, conceptualization.

## Conflicts of Interest

The authors declare no conflicts of interest.

## Supporting information


**Data S1.** Supporting Information.

**Data S2.** Supporting Information.

**Data S3.** Supporting Information.

**Data S4.** Supporting Information.

**Data S5.** Supporting Information.

**Data S6.** Supporting Information.

**Data S7.** Supporting Information.

## Data Availability

The metabolomic data and metadata have been deposited at the “Recherche Data Gouv” repository portal. Other data will be made available on request.
