# Supplementary material for: Microplastics in the Soil at Sub‐Toxic Concentrations Cause Metabolic Changes Decreasing Fungal Pathogen Susceptibility in Arabidopsis thaliana
Source: Physiol Plant. 2025 Jun 9;177(3):e70312. doi: 10.1111/ppl.70312 (PMC12147067; doi:10.1111/ppl.70312)
Supplement: Supplementary file 1 — Data S1. Supporting Information. [file PPL-177-e70312-s006.pdf]

Figure S1. Necrotic lesion diameter after 48 hours from *B. cinerea* inoculation in leaves of *A. thaliana* plants grown for three weeks in absence (C) or in presence of PET- or PVC-MPs at different concentrations (0.2 % and 0.5 % w/w). *a)* inoculation with 1×10^6 conidia ml^{-1}; *b)* inoculation with 5×10^5 conidia ml^{-1}. Values are mean of 8 replicates ± standard deviation; lower case letters indicate significant differences among the sample means (at least $p < 0.05$).

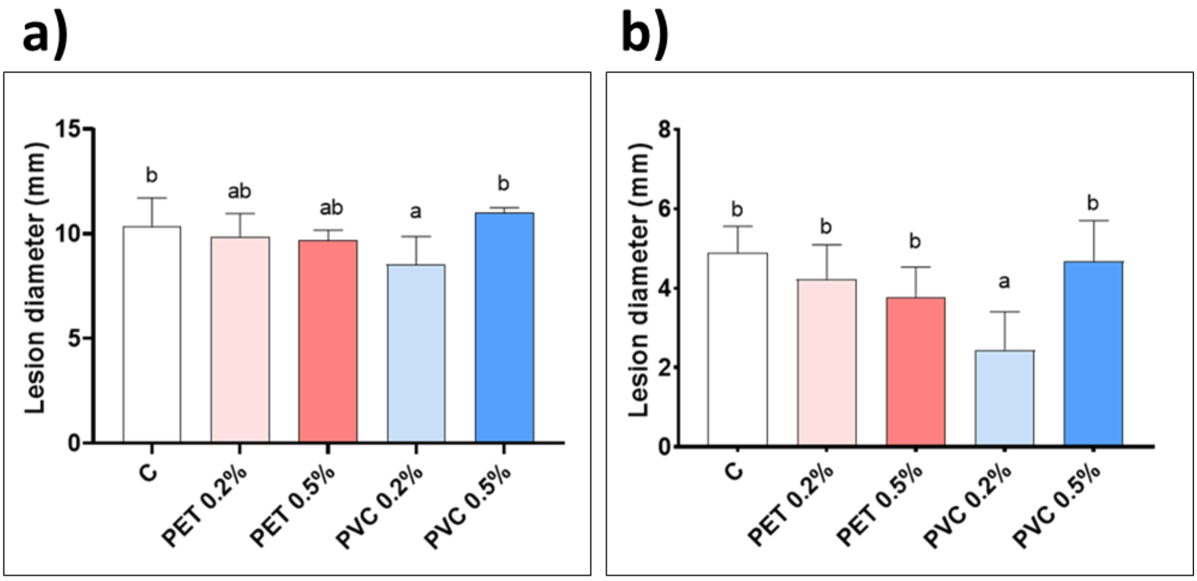

Figure S2. Confocal images showing ROS (in green) after 60 min of cerato-platanin (CP) exposure in leaves of *A. thaliana* plants grown for three weeks in absence (C) or in presence of PET- or PVC-MPs at different concentrations (0.2 % and 0.5 % w/w). Two representative leaves per treatment are shown; H_2O indicates the negative control, that is absence of cerato-platanin (CP) exposure.

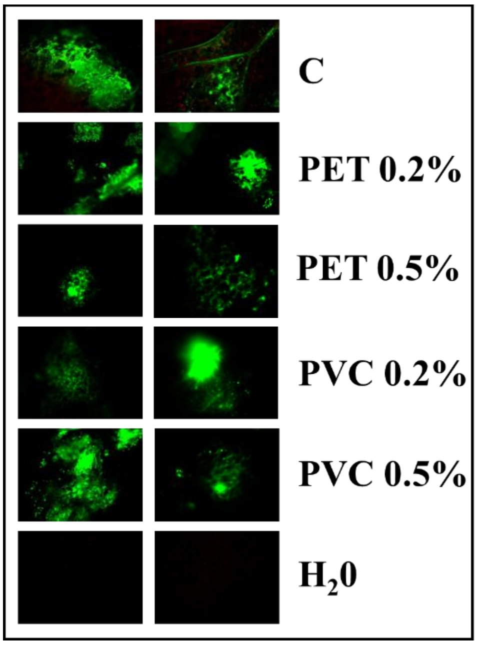

Figure S3. Venn diagram illustrating the comparison of MFs with VIP>1 in both PLS-DA components across the three different conditions: H_2O as control, cerato-platanin (CP) elicitation and *B. cinerea* infection. The histograms below indicate the size of each group, while the graph further down illustrates the number of MFs shared by one; two or three groups.

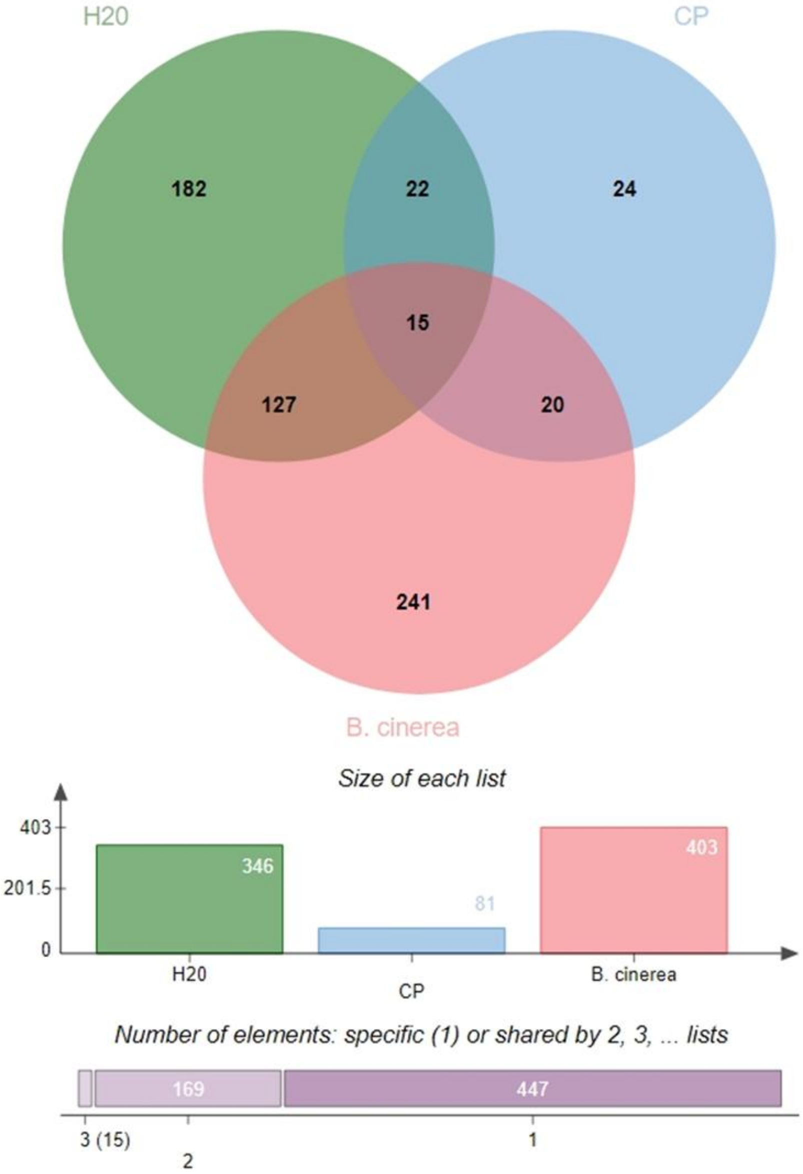

Figure S4. Heatmaps showing the different accumulation of MFs with VIP>1 in both PLS-DA components across the three different conditions, specifically H_2O as control (*a*), cerato-platanin (CP) elicitation (*b*) and *B. cinerea* infection (*c*). Colour scale indicates lower (blue) or higher (red) accumulation. Microplastic treatments and clusters are identified in the legend on the right.

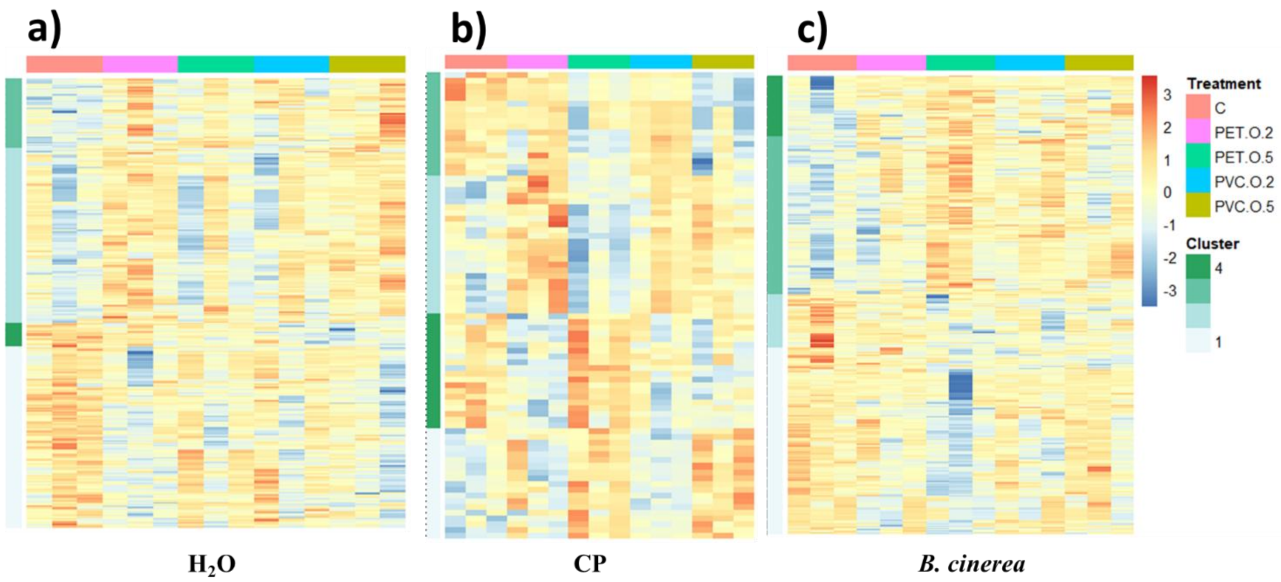

Figure S5. Co-accumulation networks (r > 0.9) of MFs with VIP>1 in both PLS-DA components across two of the three different conditions, specifically H_2O (*a*) and *B. cinerea* infection (*b*). Different colours represent distinct metabolic categories, as indicated in the legend. The shape and size of the MFs within the networks are based on the previously calculated log_2FC from the pairwise comparison of PET 0.5% vs C. The minimum and maximum log_2FC values used for scaling the sizes are indicated above the graphs.

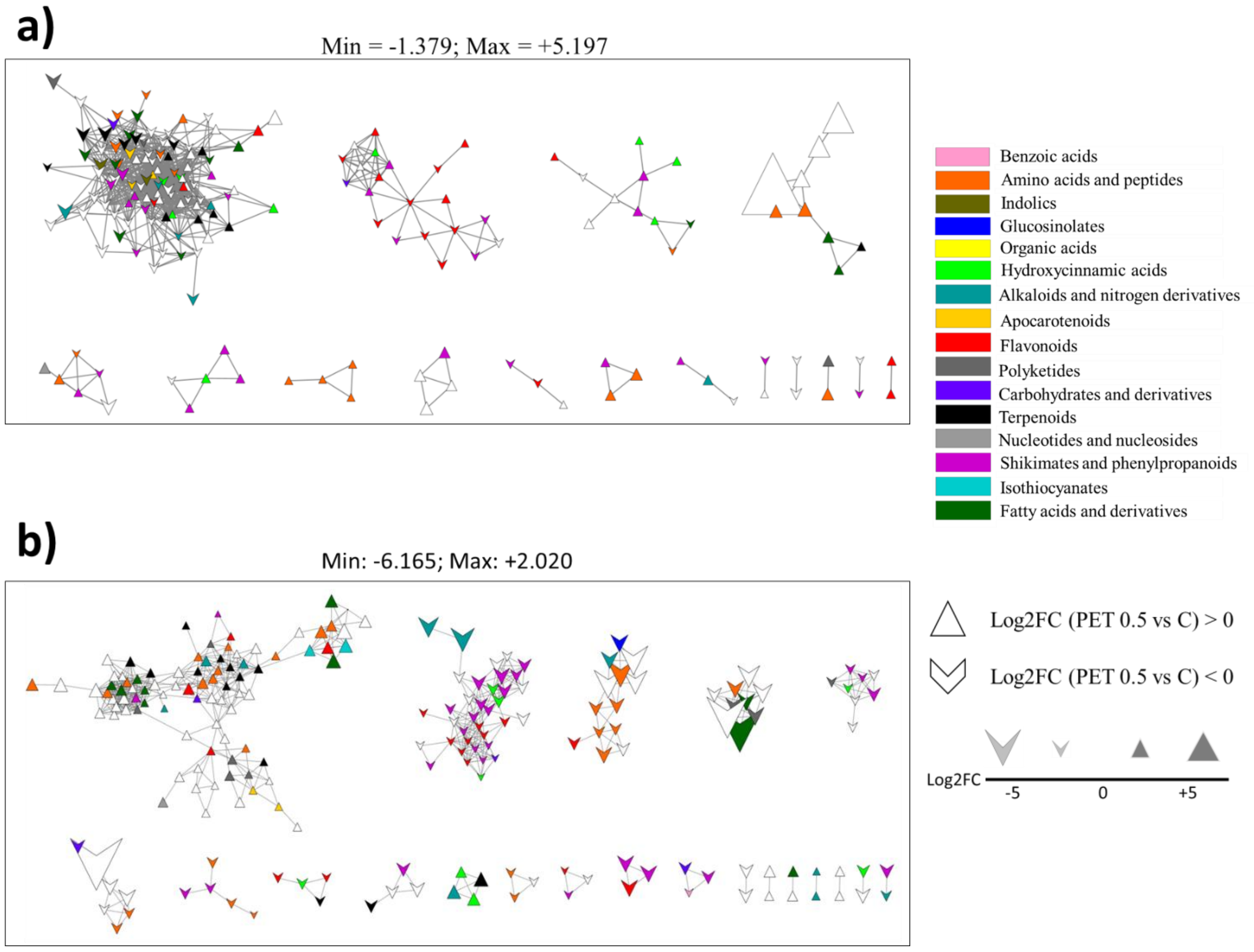

Figure S6. Boxplots, putative chemical structure, molecular formula and annotation confidence level of camalexin (*a*,*b*), oxidized glutathione (*c*,*d*) and ascorbic acid (e,*f*) across all experimental conditions. Accumulation values (3 replicates per experimental condition) were normalized and expressed as a percentage in relation to the sample exhibiting the highest accumulation value (i.e. 100%).

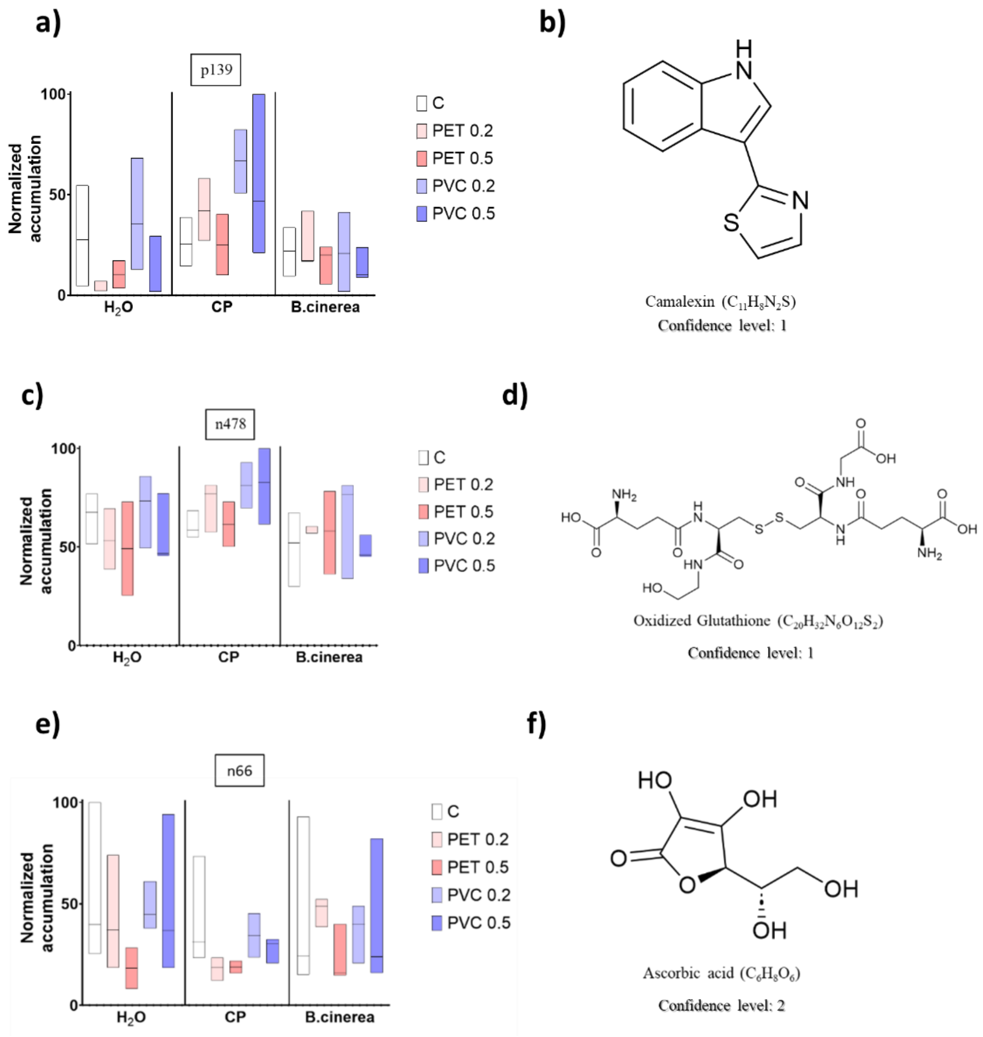

Table S1. Main abbreviations used in the manuscript and their respective definitions.

Abbreviation	Definition
**MPs**	Microplastics
**PET**	Polyethylene terephthalate
**PVC**	Polyvinyl chloride
**PAMP**	Pathogen-Associated Molecular Pattern
**ROS**	Reactive Oxygen Species
**MDA**	Malondialdehyde
**PLS-DA**	Partial Least Square Discriminant Analysis
**VIP**	Variable Importance in Projection
**PCA**	Principal Component Analysis
**MFs**	Metabolic Features
**DAMFs**	Differentially Accumulated Metabolic Features

Table S2. Selected OJIP-test parameters and relative definition according to Dainelli et al. (2024).

OJIP-test parameter	Definition
$\mathbf{F_0}$	Minimum fluorescence value at 20 µs
$\mathbf{F_M}$	Maximum fluorescence value at plateau
$\mathbf{F_V = F_M - F_0}$	Variable PSII fluorescence
$\mathbf{V_t = (F_t - F_0)/(F_M - F_0)}$	Relative variable fluorescence at time t (V_J at 2 ms, V_I at 30 ms)
$\mathbf{\Phi P_o = F_v/F_m}$	Estimate of PSII maximum quantum yield
$\mathbf{\Psi ETo = 1\text{-}V_j}$	Probability of which a PSII-trapped electron is transferred beyond reduced Q_A, or estimate of the oxidized-plastoquinone size
$\mathbf{\Psi REo = 1\text{-}V_i}$	Probability of which a PSII trapped electron is transferred from reduced Q_A to PSI final acceptors, or estimate of the relative size of the pool of PSI end-electron acceptors
**Sm**	Normalized area above OJIP transient, proportional to the number of electron carriers per electron transport chain
**ABS/RC**	Flux of light absorption per active reaction center, or apparent antenna size of an active PSII
$\mathbf{Pi_{abs}}$	Potential ability for energy conservation, from absorbed photons in PSII to the reduction of electron acceptors.

Table S3. Element concentration (mg g^{-1} and μg g^{-1} for K, Ca, Mg and Zn, Mn, Fe, Cu, respectively) in shoots of *A. thaliana* plants grown for three weeks in absence (C) or in presence of PET- or PVC-MPs at different concentrations (0.2 % and 0.5 % w/w). Values are mean of 8 replicates ± standard deviation. Lower case letters indicate significant differences among control and treated samples (at least $p < 0.05$).

**Element concentration**	**Treatments**				
	**C**	**PET 0.2%**	**PET 0.5%**	**PVC 0.2%**	**PVC 0.5**
**K**	49.1 ± 1.9 **a**	44.4 ± 9.8 **a**	47.9 ± 1.9 **a**	49.7 ± 6.4 **a**	48.1 ± 1.8 **a**
**Ca**	76.8 ± 1.8 **b**	73.6 ± 5.3 **ab**	75.3 ± 2.4 **b**	75.8 ± 2.5 **b**	70.3 ± 1.2 **a**
**Mg**	5.9 ± 0.2 **b**	5.4 ± 0.6 **ab**	5.6 ± 0.3 **ab**	5.5 ± 0.7 **ab**	5.1 ± 0.2 **a**
**Zn**	202.1 ± 24.6 **c**	183.8 ± 18.0 **bc**	164.6 ± 16.0 **ab**	175.9 ± 10.3 **ab**	155.8 ± 10.7 **a**
**Mn**	36.3 ± 13.7 **bc**	41.0 ± 21.5 **c**	19.2 ± 3.2 **a**	17.8 ± 3.8 **a**	21.0 ± 2.4 **ab**
**Fe**	159.1 ± 53.6 **a**	154.9 ± 47.7 **a**	155.4 ± 35.9 **a**	157.6 ± 41.1 **a**	168.9 ± 49.0 **a**
**Cu**	8.9 ± 2.0 **a**	7.0 ± 2.7 **a**	8.4 ± 2.2 **a**	8.1 ± 1.9 **a**	8.4 ± 1.70 **a**

Table S4. Pair-wise PERMANOVA results for each condition: H_2O as control (*a*), cerato-platanin (CP) elicitation (*b*) and *B. cinerea* infection (*c*) All the possible MP-stress combinations are presented; P-value is based on 999 permutations.

a) **Pair-wise PERMANOVA results**			**H_2O**	
	**F**	**R^2**	**P-value**	**Adjusted P-value**
**C vs PET 0.2%**	6.677	0.625	0.100	0.500
**C vs PET 0.5%**	1.132	0.248	0.300	0.500
**C vs PVC 0.2%**	1.184	0.228	0.400	0.571
**C vs PVC 0.5%**	6.654	0.625	0.100	0.500
**PET 0.2% vs PET 0.5%**	1.999	0.333	0.300	0.500
**PET 0.2% vs PVC 0.2%**	1.179	0.228	0.500	0.625
**PET 0.2% vs PVC 0.5%**	0.001	0.001	1.000	1.000
**PET 0.5% vs PVC 0.2%**	0.061	0.015	0.900	1.000
**PET 0.5% vs PVC 0.5%**	2.009	0.334	0.300	0.500
**PVC 0.2% vs PVC 0.5%**	1.198	0.230	0.300	0.500
b) **Pair-wise PERMANOVA results**			**CP**	
	**F**	**R^2**	**P-value**	**Adjusted P-value**
**C vs PET 0.2%**	7.861	0.663	0.100	0.143
**C vs PET 0.5%**	6.185	0.607	0.100	0.143
**C vs PVC 0.2%**	3.553	0.470	0.200	0.222
**C vs PVC 0.5%**	11.982	0.750	0.100	0.143
**PET 0.2% vs PET 0.5%**	11.250	0.738	0.100	0.143
**PET 0.2% vs PVC 0.2%**	1.507	0.274	0.300	0.300
**PET 0.2% vs PVC 0.5%**	4.169	0.510	0.200	0.222
**PET 0.5% vs PVC 0.2%**	9.982	0.714	0.100	0.143
**PET 0.5% vs PVC 0.5%**	5.470	0.578	0.100	0.143
**PVC 0.2% vs PVC 0.5%**	7.155	0.641	0.100	0.143
a) **Pair-wise PERMANOVA results**			***B.cinerea***	
	**F**	**R^2**	**P-value**	**Adjusted P-value**
**C vs PET 0.2%**	1.819	0.313	0.200	0.250

**C vs PET 0.5%**	8.250	0.673	0.100	0.250
**C vs PVC 0.2%**	4.465	0.527	0.100	0.250
**C vs PVC 0.5%**	2.818	0.413	0.200	0.250
**PET 0.2% vs PET 0.5%**	3.756	0.484	0.200	0.250
**PET 0.2% vs PVC 0.2%**	1.836	0.315	0.200	0.250
**PET 0.2% vs PVC 0.5%**	1.420	0.262	0.400	0.444
**PET 0.5% vs PVC 0.2%**	3.603	0.474	0.100	0.250
**PET 0.5% vs PVC 0.5%**	4.873	0.549	0.100	0.250
**PVC 0.2% vs PVC 0.5%**	0.345	0.079	0.700	0.700
