# Supplementary material for: Microplastics in the Soil at Sub‐Toxic Concentrations Cause Metabolic Changes Decreasing Fungal Pathogen Susceptibility in Arabidopsis thaliana
Source: Physiol Plant. 2025 Jun 9;177(3):e70312. doi: 10.1111/ppl.70312 (PMC12147067; doi:10.1111/ppl.70312)
Supplement: Supplementary file 2 — Data S2. Supporting Information. [file PPL-177-e70312-s004.pdf]

UPLC–MS/MS data processing

mzXML data processing, mass detection, chromatogram building, deconvolution, samples alignment, and data export, were performed using MZmine 3.6.0 software (http://mzmine.github.io/) for both positive and negative data files. The Automated Data Analysis Pipeline (ADAP) chromatogram builder (Myers et al., 2017) method was used with a minimum group size of scan 4, a group intensity threshold of 5000 in pos mode, 100 in neg mode, a minimum highest intensity of 5500 in pos mode, 150 in neg mode and m/z tolerance of 10 ppm for both modes. Deconvolution was performed with the ADAP wavelets algorithm using the following setting: S/N threshold 8, peak duration range = 0.01-2 min in pos mode, 0.01-0.5 min in neg mode RT wavelet range 0.1-0.2 min for both modes, MS2 scan were paired using a m/z tolerance range of 0.01 Da and RT tolerance of 0.1 min. Then, isotopic peak grouper algorithm was used with a m/z tolerance of 10 ppm and RT tolerance of 0.2min for both modes. All the peaks were filtered using feature list row filter keeping only peaks with MS2 scan. The alignment of samples was performed using the join aligner with an m/z tolerance of 10 ppm, a weight for m/z and RT at 1, a retention time tolerance of 0.1 min for both modes.

References

Myers O. D., Sumner S. J., Li S., Barnes S. & Du X. (2007) One step forward for reducing false positive and false negative compound identifications from mass spectrometry metabolomics data: New algorithms for constructing extracted ion chromatograms and detecting chromatographic peaks. *Analytical Chemistry* 89, 8696–8703
